# Phantom benchmarking for quality assurance of synthetic computed tomography in cranial magnetic resonance-only radiotherapy^☆^

**DOI:** 10.1016/j.phro.2026.101062

**Published:** 2026-08-14

**Authors:** Bernd-Niklas Axer, Johann Brand, Lucas Pieper, Florian Putz, Johanna Lott, Christoph Dickmann, Rainer Fietkau, Stefanie Corradini, Christoph Bert, Juliane Szkitsak

**Affiliations:** a Department of Radiation Oncology, University Hospital Erlangen, Friedrich-Alexander-University Erlangen-Nürnberg (FAU), Germany; b Comprehensive Cancer Center Erlangen-EMN (CCC ER-EMN), Erlangen, Germany; c Comprehensive Cancer Center Alliance WERA (CCC WERA), Erlangen, Germany; d Bavarian Cancer Research Center (BZKF), Erlangen, Germany; e Siemens Healthineers AG, Forchheim, Germany

**Keywords:** Synthetic computed tomography, Quality assurance, Head phantom

## Abstract

Background and Purpose: Synthetic computed tomography (sCT) from magnetic resonance imaging (MRI) enables computed tomography (CT)-free cranial radiotherapy planning, yet no consensus exists on which head phantoms are usable for sCT quality assurance (QA). This study benchmarked head phantoms to identify the most promising candidates for cranial sCT QA and the design characteristics associated with favorable sCT-to-conventional CT agreement.

Materials and Methods: Ten head phantoms underwent clinical MRI (1.5 T) and CT imaging. Two vendor-provided sCT algorithms (2D slice-based, 3D volume-based) were applied to identical input. sCT and CT were compared using Dice similarity coefficient (DSC), 95th-percentile Hausdorff distance (HD95), CT number mean error (ME) within both a self-mask (sCT-derived) and a common-mask (CT-derived), and percentage dose deviations (%ΔD_2%_, %ΔD_98%_, %ΔD_mean_) across six beam configurations. Metrics were interpreted against a priori three-tier acceptance thresholds.

Results: Phantoms without internal skull-equivalent structures showed bone DSC ≤ 0.001 and common-mask bone ME≤ −1100 HU. Phantoms with skull-equivalent anatomy achieved bone DSC of 0.24–0.71, bone HD95 of 4.2–24.4 mm, and self-mask bone ME between −95 and +470 HU across both algorithms. Common-mask bone ME in skull-containing phantoms ranged from −198 to −1115 HU across both algorithms. Soft-tissue ME stayed within ±120 HU across skull-containing phantoms for both masks. Average absolute |%ΔD_mean_| was 3.2% (2D) and 3.7% (3D) without significant inter-algorithm difference (*p* = 0.49).

Conclusions: Internal skull-equivalent anatomy is a necessary but not sufficient design characteristic; low-susceptibility and MR-compatible materials proved essential. Joint evaluation of geometric, CT number, and dose agreement was required to rank phantom performance. No phantom met all criteria simultaneously.

## Highlights

• Ten head phantoms are benchmarked for synthetic computed tomography quality assurance.
• Skull-free phantoms show common-mask bone errors below −1100 Hounsfield units.
• Skull-containing phantoms show self-mask bone errors of −95 to 470 Hounsfield units.
• Best performing phantom reaches mean dose deviation below 2% in the target volume.
• Findings guide phantom design for routine synthetic computed tomography monitoring.

## Graphical abstract

**Figure:**
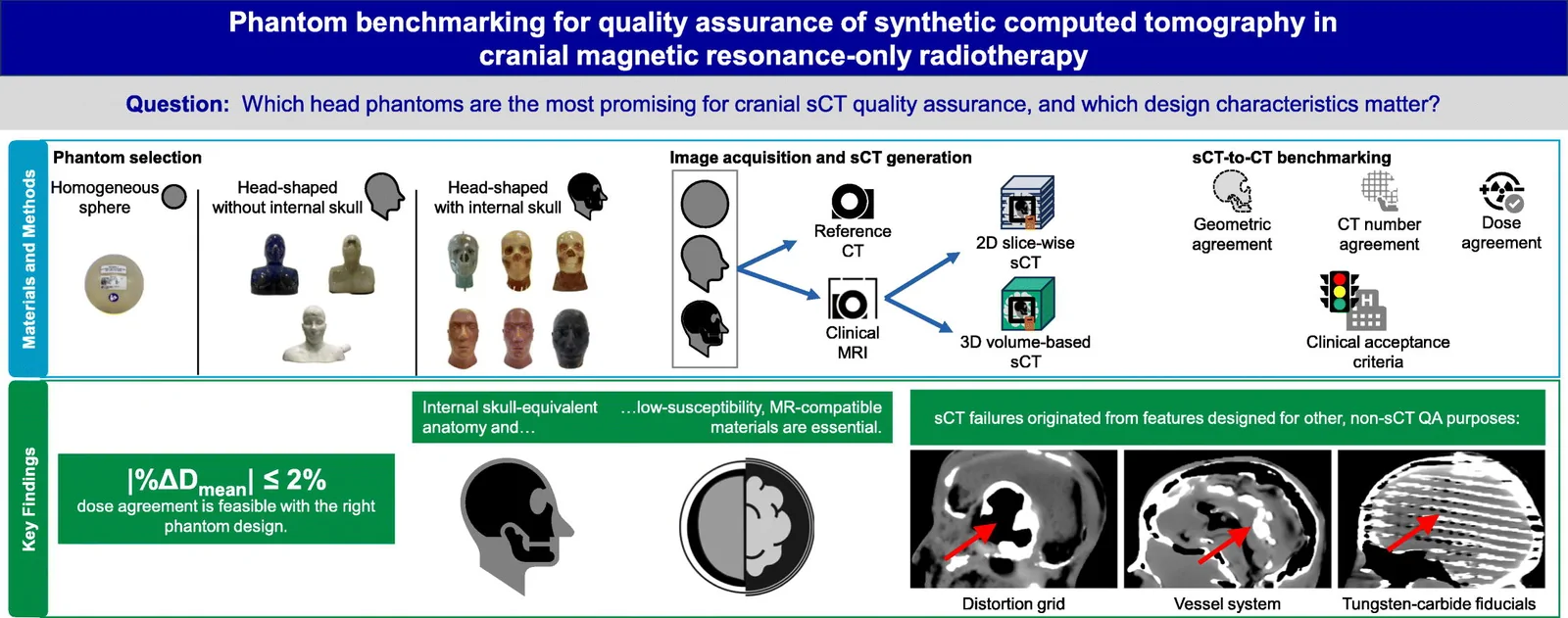

## 1 Introduction

Magnetic resonance imaging (MRI) plays a central role in cranial radiotherapy planning because of its superior soft-tissue contrast [1]. In magnetic resonance (MR)-only workflows, synthetic computed tomography (sCT) images derived from MRI data replace conventional computed tomography (CT) for dose calculation and image guidance at the linear accelerator. This approach eliminates CT-MRI registration uncertainty [2]. For cranial stereotactic radiotherapy, where targeting accuracy within 1–2 mm is expected, sCT images must provide accurate geometric representation and CT number fidelity [3], [4]. Failure-mode analyses have therefore identified sCT generation as a risk-relevant step in MR-only pathways, highlighting the need for dedicated quality assurance (QA) [5].

At present, however, no consensus standard exists for sCT-specific QA. Clinical implementations have relied on general MRI simulation QA frameworks that ensure basic MRI quality parameters, but do not define sCT-specific acceptance criteria for MR input and the generated sCT itself [6], [7]. Recent recommendations have called for dedicated sCT QA but have noted the absence of validated commercial solutions [8]. Current practice shows substantial variability, ranging from mean error (ME) checks to cone-beam CT recalculation and visual reviews [1]. A common limitation across these approaches is the lack of a CT ground truth in routine MR-only workflows, which prevents direct comparison of sCT outputs against a fixed reference [8]. Reproducible objects that can be imaged repeatedly and compared with a conventional CT baseline (reference CT, rCT) could in principle fill this role.

Anthropomorphic phantoms have been proposed as a practical implementation of this concept [8], [9], [10]. They enable standardized testing under clinically relevant conditions and have been recommended for end-to-end QA, distortion characterization, and image-quality benchmarking [1], [6], [7]. For sCT QA specifically, phantoms may provide a physical reference for assessing three complementary dimensions of performance: geometric agreement, CT number fidelity, and dose consistency [11], [12]. However, currently available head phantoms were not primarily developed for cranial sCT QA in MR-only radiotherapy, and no systematic comparison has established which designs are suitable for this purpose [8]. In addition, because current sCT algorithms are predominantly trained on patient anatomy, the extent to which phantom-derived metrics translate to patient performance remains an open question.

This study therefore benchmarked a set of head phantoms spanning different levels of anatomical and material complexity in a cranial MR-only workflow focusing on sCT-based dose calculation. Two vendor-provided sCT algorithms were applied to identical MRI input and compared with rCT using geometric, CT number, and dose metrics. We hypothesized greater agreement for phantoms with internal skull-equivalent structures. The primary aim was to identify the most promising candidates for cranial sCT QA and the phantom design characteristics associated with favorable sCT-to-rCT agreement.

## 2 Materials and methods

### 2.1 Phantom selection

A structured review of head phantoms based on manufacturer catalogs, product documentation, and vendor communication identified fourteen candidates. Inclusion required (1) MRI imageability, (2) external dimensions comparable to an adult head, compatible with standard cranial setup positioning, and (3) long-term material stability under repeated handling. Four phantoms were excluded: one for insufficient MRI imageability, two for incompatible external dimensions, and one for limited stability (Supplementary Material S1). The ten included phantoms (Table 1) span three complexity levels: a homogeneous sphere (A), included as a deliberately simplified geometric reference, head-shaped phantoms without internal skull (B–D), and skull-containing phantoms with bone-equivalent materials (E–J).

### 2.2 Image acquisition and synthetic computed tomography generation

All MRI examinations were performed on a clinically used 1.5 T MAGNETOM Sola (Siemens Healthineers AG, Forchheim, Germany) using a diagnostic 20-channel BioMatrix Head/Neck 20 coil (Siemens Healthineers AG). A T1-weighted VIBE-Dixon sequence (volumetric interpolated breath-hold examination with two-point water-fat separation, Siemens Healthineers AG), which is the vendor-specified input for sCT generation, was applied uniformly (slice thickness 0.8 mm, echo times TE₁/TE₂ = 2.39/4.77 ms, repetition time 6.7 ms). A homogeneous bottle phantom adjacent to each specimen stabilized signal reception and was excluded from analysis.

**Table 1:** Overview of the head phantoms included in the primary analysis. For each phantom, an image, model name, manufacturer, primary composition, and intended original use are provided. Abbreviations: CT = Computed Tomography, ICRU = International Commission on Radiation Units and Measurements, MRI = Magnetic Resonance Imaging, QA = Quality Assurance, RT = Radiotherapy.

Phantom	Image	Name	Manufacturer	Primary composition	Intended original use
A		Spherical Phantom D165	Siemens Healthineers AG, Germany	Homogeneous silicone oil-based medium	MRI QA
B		MR HEAD AGAR	Siemens Healthineers AG, Germany	Homogeneous aqueous agar-based gel	MRI sequence development
C		MR HEAD BLUE	Siemens Healthineers AG, Germany	Homogeneous mineral oil-based medium	MRI sequence development
D		MR HEAD WHITE	Siemens Healthineers AG, Germany	Proprietary homogeneous synthetic medium	MRI sequence development
E		MR Distortion and Image Fusion Head Phantom (Model 603-GS)	Sun Nuclear, a Mirion Medical Company, USA	Plastic-based trabecular bone substitute; proprietary water-based polymer soft-tissue	MRI geometric distortion; verify CT/MR image fusion and deformable registration [23]
F		ALDERSON Phantom Patient	Radiology Support Devices, Inc., USA	Tissue-equivalent soft-tissue; polymer bone substitute (per ICRU-44)	Whole-body dosimetry and RT QA across external beam modalities, film/ion-chamber measurements [24]
G		ExacTrac Dynamic Cranial Verification Phantom (Original, with tungsten-carbide spheres)	Brainlab SE, Germany	Bone-equivalent skull & cervical vertebrae; tissue-equivalent outer material; tungsten-carbide spheres	QA and training for ExacTrac Dynamic cranial workflows [25]
H		ExacTrac Dynamic Cranial Verification Phantom (Adapted, without tungsten-carbide spheres)	Brainlab SE, Germany	Bone-equivalent skull & cervical vertebrae; tissue-equivalent outer material	Study use only, based on [25]
I		Adult Head (Dynamic)	True Phantom Solutions, Canada	Ultra-soft polyurethane-based brain parenchyma; 3-layer bone-like skull, fillable internal blood vessels	Multi-modal imaging phantom for training, QA and motion/flow simulations [26]
J		Adult Head (Static)	True Phantom Solutions, Canada	Urethane-based soft resin; ceramic-reinforced epoxy-based synthetic bone	Medical imaging of foreign static objects such as small bone pieces, shrapnel, bullets and any other metal or non-metal targets [27]

sCT images were generated from the Dixon datasets in syngo.via RT Image Suite (Siemens Healthineers AG) as in clinical routine: a two-dimensional slice-wise model (2D sCT, version VB60) and a three-dimensional volume-based neural network (3D sCT, version VC10, undergoing clinical clearance at the time of the study). MR input was transferred directly from the scanner to the sCT algorithms without user-side intensity normalization, bias-field correction, or other preprocessing; any vendor-internal preprocessing was applied by the sCT software. Both algorithms were applied to the same input volumes using vendor default settings. Neither algorithm was re-trained, fine-tuned, or calibrated on local or phantom data.

rCT images were acquired on a SOMATOM go.Open Pro (Siemens Healthineers AG) using a clinical cranial protocol (120 kV, 45 mA, 1.0 mm slice thickness, iterative reconstruction strength S3). The sharp head Hr60 kernel was chosen to enhance edge sharpness and separation at air-bone and bone-soft-tissue interfaces, thereby supporting quantitative geometric assessment.

### 2.3 Segmentation and image preprocessing

Image processing used 3D Slicer 5.8.0 [13] with the SlicerRT extension [14]. Semi-automatic threshold-based segmentation produced binary masks for soft-tissue (−150 to 150 Hounsfield units (HU)) and bone (> 150 HU, following Singhrao et al. [15]). Segmentations were visually inspected; non-anatomical structures from immobilization were removed manually (Supplementary Material S2). Rigid registration of each sCT to its corresponding rCT was performed with the BRAINSFit module (Mattes mutual information, linear interpolation, sampling fraction 0.2%, ≤ 1500 iterations, step length 0.05–0.001, no masking); remaining parameters were left at default.

### 2.4 Geometric and CT number agreement

Geometric agreement between rCT and sCT segmentations was assessed using the Dice similarity coefficient (DSC) [16], [17] and the 95th-percentile Hausdorff distance (HD95) [18], [19], within SlicerRT [14]. CT number values were evaluated using two complementary masking approaches. The self-mask was the segmentation obtained from the sCT. The common-mask was the segmentation obtained from the rCT. Each mask was applied to both images to sample mean HU within the same set of voxels. The self-mask therefore evaluates CT number agreement only within voxels the sCT itself labels as the respective tissue, whereas the common-mask anchors the evaluation to the rCT-defined tissue and additionally captures misclassifications where the sCT mislabels tissue voxels. For each mask, the ME was computed as ME = mean(HU_sCT_) − mean(HU_rCT_), where positive values indicate overestimation of tissue density by the sCT algorithm.

### 2.5 Dose calculation and analysis

Dose calculation used RayStation (version 12 A, RaySearch Laboratories, Stockholm, Sweden) with the collapsed cone convolution algorithm. On the rCT, a spherical planning target volume (PTV, 10 mm diameter) was placed at the geometric center of the brain. Four identical control volumes were positioned 50 mm to the anterior, posterior, left, and right of the PTV to probe directional dose stability [20].

A single-fraction plan (6 MV) comprising six beam configurations was created on the rCT: a four-field coplanar 3D conformal arrangement combining anterior-posterior and left-right beams (APLR, 500 monitor units (MU)), four single-field 3D conformal beams from the anterior (AP), posterior (PA), left-lateral (LR), and right-lateral (RL) directions (1500 MU each), and a single-arc volumetric modulated arc therapy (VMAT) plan (gantry 181°–179°, 1424 MU). The APLR and single fields were delivered at fixed MU values to provide a defined MU input for each directional probe. The VMAT was optimized with a prescription dose of 10.0 Gy to the PTV.

The rCT plan was rigidly transferred to each sCT without re-optimization. All regions of interest were propagated using the same transformation (Supplementary Material S3). For the PTV and every control volume, three dose-volume histogram (DVH) metrics (D_2%_, D_98%_, and D_mean_) were extracted on both rCT and sCT. As defined in Eq. (1), for each metric X, the signed percentage difference %ΔX was computed as:

(1) $$\%\mathrm{\Delta X}=100\times \left(X\_\mathrm{sCT}-X\_\mathrm{rCT}\right)/X\_\mathrm{rCT}$$

Absolute values (|%ΔX|) were used for aggregated results across phantoms, algorithms, or beam configurations to prevent cancellation of opposing deviations; signed %ΔX was retained for per-phantom and per-configuration reporting.

### 2.6 Statistical analysis

These comparisons tested whether the two vendor algorithms differ systematically in phantom-based sCT-to-rCT agreement. Paired metrics from the 2D and 3D sCT algorithms were compared on each geometric, CT number, and dose metric using two-sided Wilcoxon signed-rank tests [21], [22], chosen for their robustness to non-normal distributions in small samples. Statistical comparisons of bone metrics were restricted to skull-containing phantoms (E–J, *n* = 6). Bone metrics for A–D are reported descriptively as a negative control, characterizing the expected failure mode in the absence of bone-equivalent structures. Soft-tissue and dose comparisons included all ten phantoms (*n* = 10). Statistical significance was set at α = 0.05. Rank-biserial correlations (r) were reported as effect sizes, and 95% confidence intervals for median paired differences were estimated via bootstrap resampling (10,000 iterations). No multiplicity correction was applied.

**Table 2:** Overview of the acceptance thresholds applied to the geometric, CT number, and dose deviation metrics. For each metric, the acceptable (green), marginal (yellow), and unacceptable (red) thresholds and the supporting references are provided. Abbreviations: DSC = Dice Similarity Coefficient, HD95 = 95th Percentile Hausdorff Distance, HU = Hounsfield Units, ME = Mean Error, |%ΔX| = absolute value of the signed percentage difference of DVH metric X (D₂_%_, D₉₈_%_, or D_mean_), as defined in Eq. (1).

Metric	Acceptable (green)	Marginal (yellow)	Unacceptable (red)	References
Bone DSC	≥ 0.8	0.6 ≤ DSC < 0.8	< 0.6	[28], [29], [30], [31], [32], [33], [34], [35], [36]
Bone HD95 (mm)	≤ 2.0	2.0 < HD95 ≤ 5.0	> 5.0	[4], [33], [37], [38]
Bone |ME| (HU)	≤ 100	100 < |ME| ≤ 400	> 400	[30], [33], [35], [36], [39]
Soft-tissue |ME| (HU)	≤ 10	10 < |ME| ≤ 20	> 20	[33], [35], [36]
|%ΔX|	≤ 1.0	1.0 < |%ΔX| ≤ 2.0	> 2.0	[30], [31], [33], [34], [35], [36], [40], [41], [42], [43], [44]

### 2.7 Performance classification

To enable absolute interpretation, a three-tier traffic-light classification was defined a priori based on published cranial sCT validations and applicable guideline tolerances (Table 2; Supplementary Material S4). Green denotes performance consistent with reported clinical validations, yellow indicates deviations beyond typical reported ranges but within the broader envelope of accepted values, and red marks deviations beyond clinically acceptable bounds. Each combination of phantom, algorithm, and metric is represented in the corresponding heatmap (Fig. 2, Fig. 3) as a single-colored cell; per-phantom tier counts reported in the Results refer to these cells.

## 3 Results

### 3.1 Geometric agreement

**Fig. 1:**
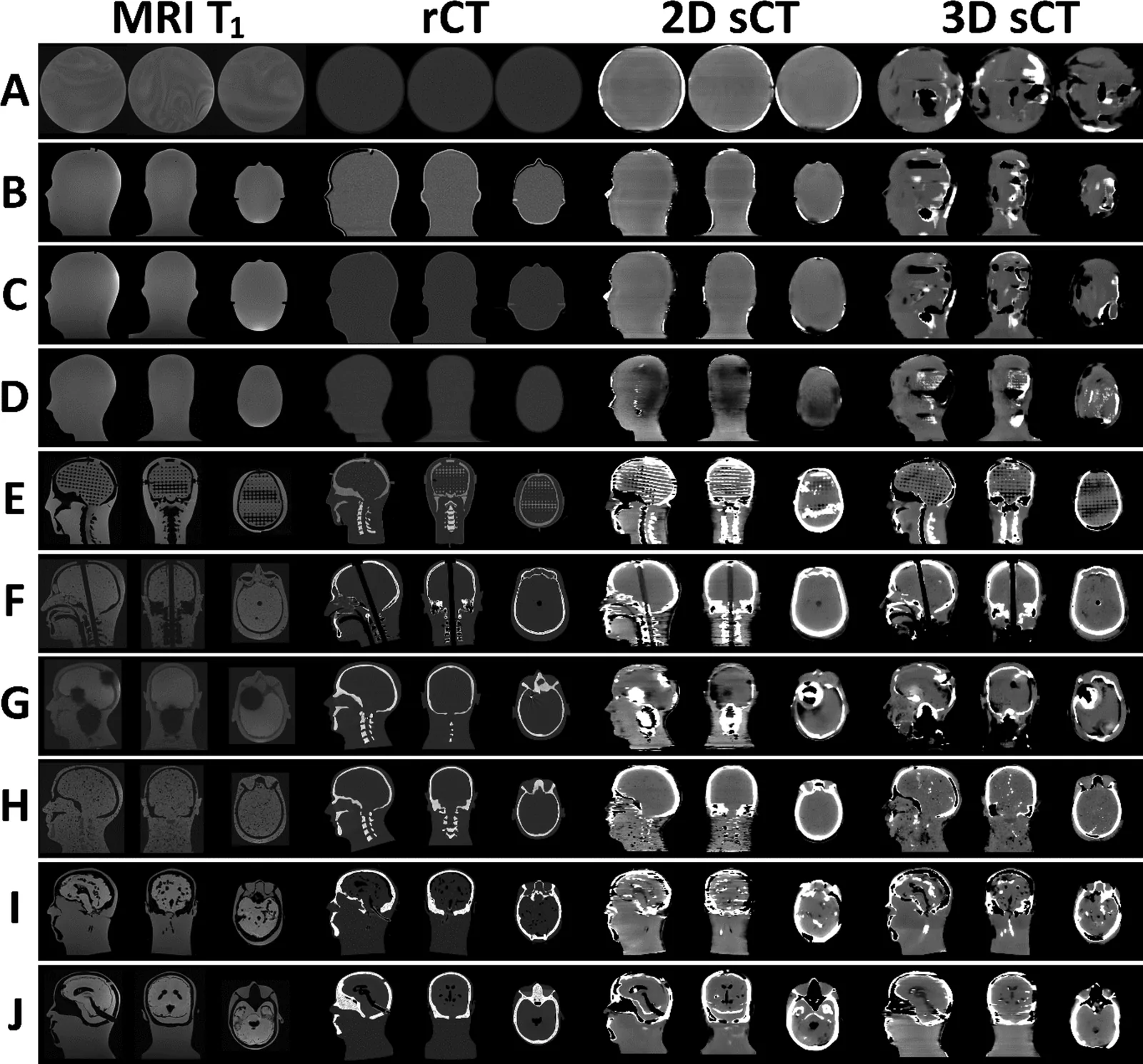
Representative sagittal, coronal, and axial slices for all ten phantoms. Columns show the MRI T1-weighted VIBE-Dixon in-phase sequence, reference CT (rCT), 2D synthetic CT (sCT), and 3D sCT. Phantoms are ordered by increasing anatomical complexity from A (homogeneous sphere) to J (anthropomorphic head with skull-equivalent structures). Abbreviations: CT = Computed Tomography, MRI = Magnetic Resonance Imaging.

**Fig. 2:**
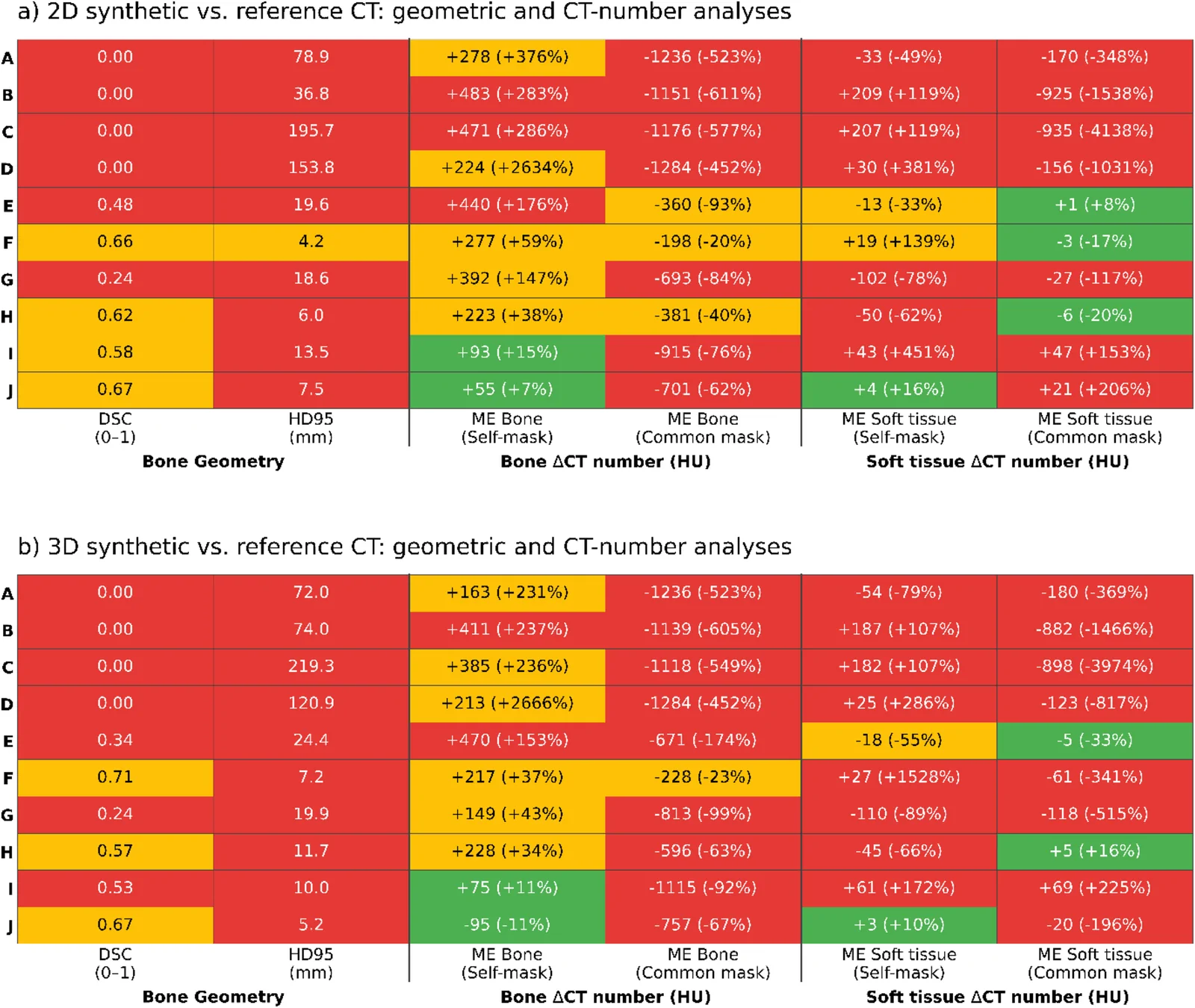
Traffic-light heatmap of segmentation geometric and CT number agreement for the (a) 2D and (b) 3D sCT. Columns show DSC, HD95, and mean CT number error (ME) evaluated within the sCT-defined mask (self-mask) and the rCT-defined mask (common-mask). ME values are reported as absolute CT number difference with relative deviation in parentheses. Color thresholds are: DSC green ≥0.8, yellow 0.6–0.8, red <0.6; HD95 green ≤2.0 mm, yellow 2.0–5.0 mm, red >5.0 mm; Bone ME green |ME| ≤ 100 HU, yellow 100–400 HU, red >400 HU; Soft tissue green |ME| ≤ 10 HU, yellow 10–20 HU, red >20 HU. See main text for mask definitions and threshold rationale. sCT = synthetic computed tomography; rCT = reference CT; DSC = Dice similarity coefficient; HD95 = 95th percentile Hausdorff distance; ME = mean error; HU = Hounsfield units. (For interpretation of the references to color in this figure legend, the reader is referred to the web version of this article.)

The sCT generation succeeded for all phantoms (Fig. 1). For skull-containing phantoms (E–J), bone DSC ranged from 0.24 (G) to 0.71 (F) and bone HD95 ranged from 4.2 mm (F) to 24.4 mm (E) across both algorithms (Fig. 2). Soft-tissue DSC remained ≥ 0.79 and soft-tissue HD95 ≤ 14.5 mm across all skull-containing phantoms.

### 3.2 CT number agreement

For phantoms without internal skull-equivalent structures (A–D), self-mask bone ME was uniformly positive (+163 to +483 HU across both algorithms), while common-mask bone ME was uniformly negative (below −1100 HU). Self-mask soft-tissue ME stayed within ±210 HU; common-mask soft-tissue ME was within ±180 HU for A and D but strongly negative for B and C (below −880 HU for both algorithms). Among skull-containing phantoms (E–J), self-mask bone ME was positive for both algorithms in all phantoms except 3D phantom J (−95 HU) and ranged from −95 to +470 HU overall. In the self-mask, phantoms I and J achieved green-tier classification for both algorithms. Common-mask bone ME was uniformly negative and ranged from −198 HU (F, smallest absolute deviation) to −1115 HU (I); no phantom reached the green tier, and five of six phantoms exceeded the red threshold for at least one algorithm. Both self-mask and common-mask soft-tissue ME stayed within ±120 HU for both algorithms, with three of six phantoms reaching the green tier in the common-mask for at least one algorithm. Algorithm-specific structural misclassifications were observed: the internal distortion grid of phantom E was segmented as bone by the 2D sCT and as air by the 3D sCT, the fillable vessel system in phantom I was misclassified as bone, and local signal voids occurred in phantom G near high-susceptibility tungsten-carbide markers.

### 3.3 Dose agreement

**Fig. 3:**
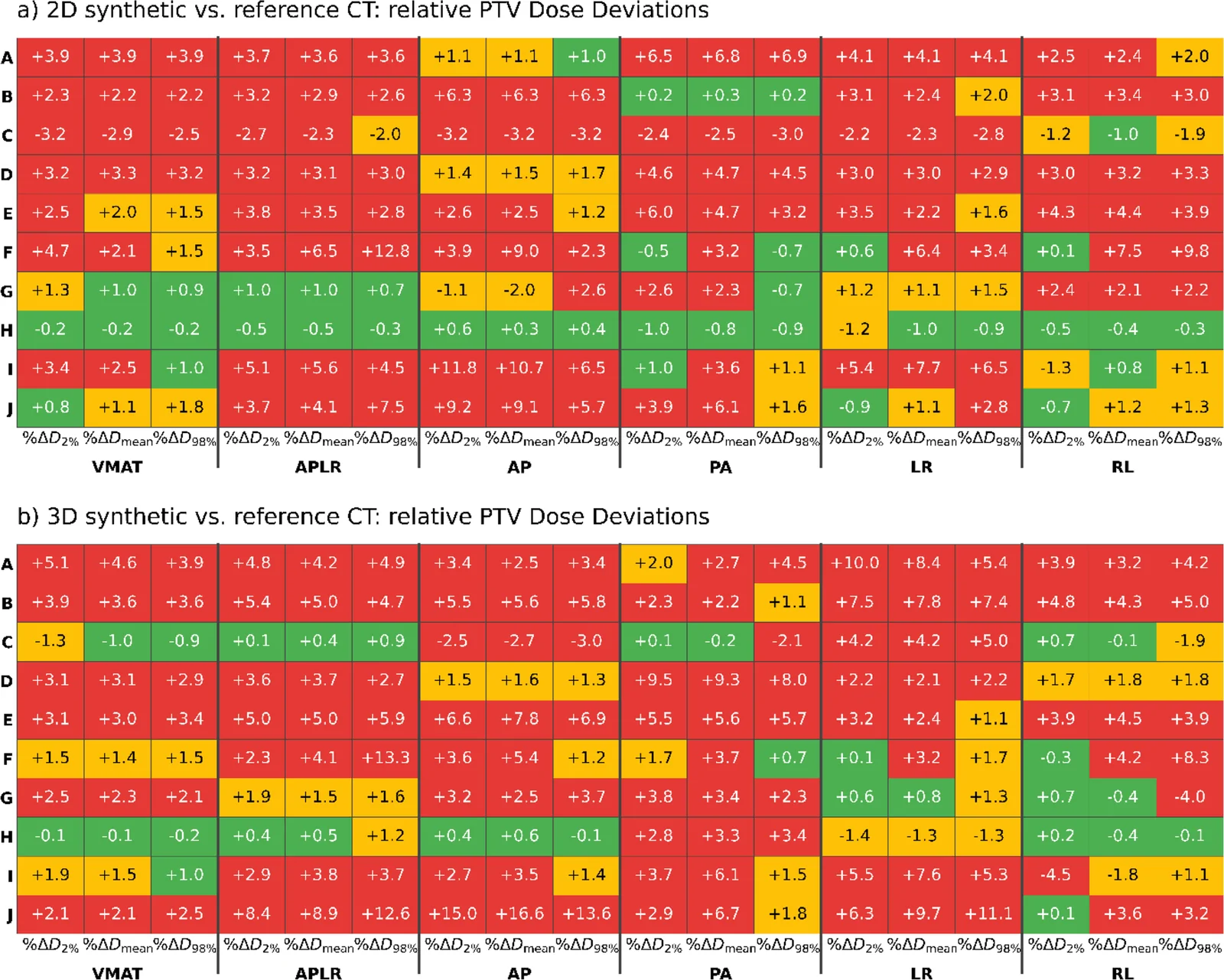
Traffic-light heatmap of relative PTV dose deviations (%) between sCT and rCT plans for the (a) 2D and (b) 3D sCT. Each cell shows %ΔD_x_ for D₂_%_, D_mean_, and D₉₈_%_ across six beam configurations (VMAT, APLR, AP, PA, LR, RL). Color thresholds are green |%ΔD_x_| ≤ 1.0%, yellow 1.0 < |%ΔD_x_| ≤ 2.0%, red >2.0%. See main text for plan definitions and threshold rationale. sCT = synthetic computed tomography; rCT = reference CT; PTV = planning target volume; VMAT = volumetric modulated arc therapy; APLR = opposed anterior-posterior/left-right field combination; AP = anterior-posterior; PA = posterior-anterior; LR = left-right; RL = right-left. (For interpretation of the references to color in this figure legend, the reader is referred to the web version of this article.)

**Fig. 4:**
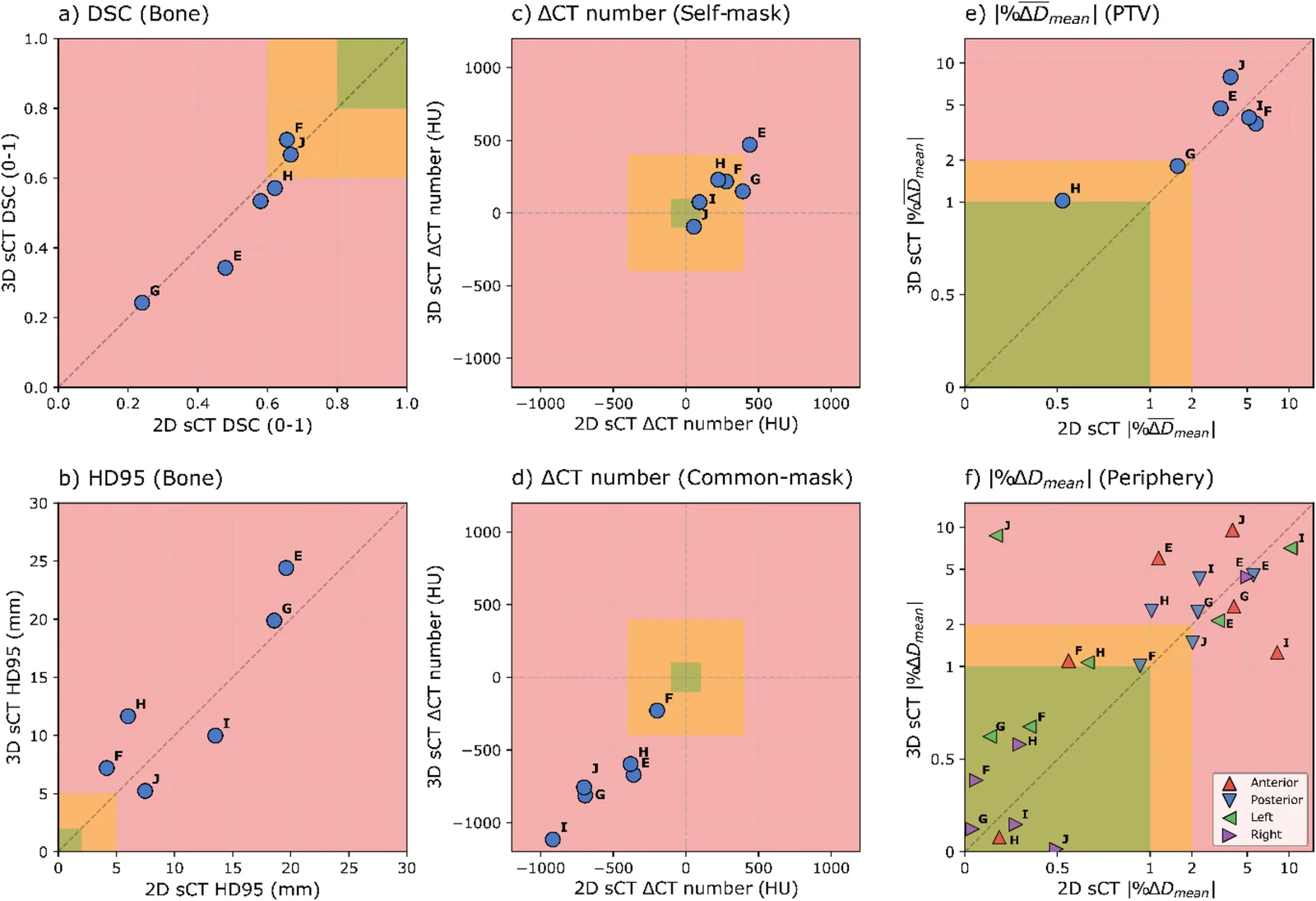
Pairwise comparison of 2D (x-axis) and 3D (y-axis) sCT performance for skull-containing phantoms (E–J). Panels show: (a) bone DSC, (b) bone HD95, (c) bone ME within the self-mask, (d) bone ME within the common-mask, (e) mean |%ΔD_mean_| across beam configurations for the central PTV, and (f) |%ΔD_mean_| for four peripheral control volumes evaluated with the ipsilateral beam arrangement. Traffic-light background shading indicates evidence-based quality tiers (see main text for threshold definitions). Dashed lines indicate equal algorithm performance (a, b, e, f) or zero deviation (c, d). Panels (e) and (f) use symmetric logarithmic scaling. See main text for mask definitions and statistical analysis. sCT = synthetic computed tomography; rCT = reference CT; DSC = Dice similarity coefficient; HD95 = 95th percentile Hausdorff distance; ME = mean error; HU = Hounsfield units; PTV = planning target volume.

%ΔD_mean_ within the central PTV was positive in 48 of 60 (2D) and 51 of 60 (3D) phantom–plan combinations (Fig. 3). For the 2D sCT, %ΔD_mean_ averaged across beam configurations ranged from −0.4% ± 0.4% (H) to +5.8% ± 2.6% (F). Only phantom H achieved predominantly green-tier classification (17 of 18 cells); phantom G showed equal distribution across all three tiers (6 green, 6 yellow, 6 red), while all remaining phantoms were classified predominantly in the red tier. For the 3D sCT, %ΔD_mean_ averaged across beam configurations ranged from +0.4% ± 1.5% (H) to +7.9% ± 5.2% (J). Phantom H achieved predominantly green-tier classification (11 of 18 cells), while all remaining phantoms were classified predominantly in the red tier. Across beam configurations, mean |%ΔD_mean_| at the central PTV across all phantoms was lowest for VMAT (2.1% for 2D, 2.3% for 3D) and highest for the AP single field (4.6% for 2D, 4.9% for 3D). In contrast, directional control volume analysis at the four peripheral control volumes showed highest |%ΔD_mean_| anteriorly (3.0% ± 3.0% for 2D, 3.5% ± 3.7% for 3D) and lowest in the right lateral direction (1.0% ± 2.0% for 2D, 0.9% ± 1.7% for 3D) (Fig. 4f).

### 3.4 Algorithm comparison

Within the common-mask, the 2D sCT showed significantly lower absolute bone ME in all six skull-containing phantoms (*p* = 0.031, *r* = 1.00, median difference −160 HU [95% confidence interval, −263 to −43]). All other comparisons were non-significant: bone DSC and HD95 (*p* = 0.44–0.69), self-mask ME for bone and soft tissue (*p* ≥ 0.69), common-mask soft-tissue ME (*p* = 0.77), soft-tissue geometric agreement (*p* = 0.084 for HD95, *p* = 0.49 for DSC), central PTV |%ΔD_mean_| (3.2% vs. 3.7%, p = 0.49), and peripheral control volumes (all *p* ≥ 0.11) (Fig. 4).

## 4 Discussion

This study benchmarked ten head phantoms in a cranial MR-only workflow by triangulating geometric agreement, CT number fidelity, and %ΔD_x_ between sCT and rCT, with the aim of identifying the most promising candidates for cranial sCT QA. Three findings structure the interpretation: (i) the presence of internal bone-equivalent anatomy was necessary but not sufficient for satisfactory sCT quality; (ii) geometric, CT number, and dose metrics each captured complementary aspects of sCT quality; and (iii) no consistent inter-algorithm advantage was detected.

The homogeneous sphere (A) and head-shaped phantoms without skull (B–D) produced systematic CT number overestimation within algorithm-generated cortical shells and severe common-mask bone ME. Skull-containing phantoms (E–J) showed non-zero DSC and clinically interpretable HD95 across algorithms. Soft-tissue ME behavior differed between the two phantom groups: phantoms A–D fell into the red tier across both masks, with particularly large deviations in B and C, whereas the majority of skull-containing phantoms reached the green tier in at least one mask. The geometric-dose dissociation was most evident in phantom J, which produced the highest bone DSC for the 2D sCT, but the largest |%ΔD_mean_| for the 3D sCT, illustrating that geometric and dose agreement can diverge. The comparison of phantom G and its tungsten-marker-free adaptation (H) isolated susceptibility artifacts as a dominant driver of residual performance differences between otherwise similar designs. Across beam configurations, single fields showed larger mean PTV |%ΔD_mean_| than the multi-field VMAT and APLR, plausibly reflecting angular averaging of directional CT number gradients in multi-field deliveries. These observations are consistent with American Association of Physicists in Medicine (AAPM) Task Group 284 [6] and the European Society for Radiotherapy and Oncology Advisory Committee on Radiation Oncology Practice (ESTRO-ACROP) guideline [7] recommendations that MR-compatible, low-susceptibility materials are essential for QA phantoms.

Three characteristics emerged as necessary conditions for sCT QA phantoms. First, internal bone-equivalent anatomy with both CT- and MR-relevant properties. Second, low-susceptibility soft-tissue and bone surrogates to avoid the signal voids and Dixon water-fat separation failures observed here. Third, MR signal characteristics within the training distribution of the patient-derived deep-learning (DL) models. Notably, the sCT failures observed here originated from features that serve the phantoms' intended non-sCT QA purposes, including the distortion grid in phantom E, the probe channel in phantom F, the fillable vessel system in phantom I, and the tungsten-carbide fiducials in phantom G. Two complementary research directions emerge from these findings: development of more anatomically realistic phantoms with MR-compatible surrogates [45], [46], and exploration of QA approaches less dependent on training-data characteristics, such as bulk-density- or atlas-based sCT methods [39], [40].

The traffic-light thresholds applied in this study should be regarded as a starting point for QA, not as a fixed acceptance standard. Operationally, the yellow tier acts as a tolerance level prompting investigation and the red tier as an action level prompting intervention before clinical use. For dose evaluation, the red tier was set at >2%, reflecting the stringent requirements of cranial stereotactic radiotherapy; for conventional fractionation with wider margins, less restrictive tiers (e.g., 3% or 5%, Supplementary Material S5) may be appropriate and should be anchored to institutional indication mix and planning margins.

Comparing the two algorithms, only the common-mask bone ME comparison reached statistical significance, favoring the 2D sCT. All other geometric, CT number, and dose comparisons were non-significant. As the comparison reflects one vendor implementation and one Dixon-based acquisition, generalization to other conditions is not supported [8].

This study has limitations. It included two algorithms from a single vendor, one MRI sequence, one scanner, and one CT reconstruction setting, thereby limiting generalizability. Using rCT as ground truth introduces protocol-specific dependencies. The statistical power was restricted by the small number of skull-containing phantoms (*n* = 6); the single significant finding should be considered exploratory. Control volumes did not include the superior-inferior axis. Phantom H was a study-use-only model rather than a commercially available phantom. Whether these phantoms detect workflow changes (e.g., sequence modifications or software updates) was not addressed and requires longitudinal studies.

For context with patient data, Masitho et al. [20] previously validated the same 2D sCT on the same scanner and sequence across 26 brain radiotherapy patients, reporting %ΔD_50%_ ≤ 2.0% across all cases. Additional brain sCT validations reported DVH-metric deviations within ±2% across commercial and research DL implementations as acceptable [30], [31], [34], [44]. Two caveats apply. First, patient-trained DL sCT models operate on input distributions that differ from phantom signal characteristics. Phantom-derived deviations may therefore under- or overestimate patient performance. Second, phantom-based QA and patient-specific dose accuracy address different questions. The former verifies stability and consistency of a reference measurement, whereas the latter reflects patient-specific anatomy and population averaging. The phantom-based dose agreement is therefore a stability benchmark for a defined QA reference object, not a predictor of patient-specific accuracy. Within this qualification, phantom H comes closest to meeting the criteria for a routine QA reference object in the present algorithm-scanner-sequence setting.

Taken together, these results demonstrate that phantom-based sCT QA is feasible, with both algorithms generating neurocranial structures on skull-containing phantoms but producing systematic failures on phantoms lacking bone-equivalent anatomy. Among the designs evaluated, a skull-containing phantom with low-susceptibility materials was the most promising candidate for periodic sCT QA, although no phantom met all criteria simultaneously. However, the substantial performance spread, driven primarily by material-related artifacts, highlights a gap between current phantom availability and clinical sCT QA requirements. Future work should extend this framework to multi-vendor, multi-sequence settings and prioritize commercially available head phantoms.

## Declaration of generative AI and AI-assisted technologies in the manuscript preparation process

During the preparation of this work the authors used Claude (Anthropic) and Microsoft Copilot (Microsoft) in order to improve the English language and readability. After using these tools/services, the authors reviewed and edited the content as needed and take full responsibility for the content of the published article.

## CRediT authorship contribution statement

**Bernd-Niklas Axer:** Writing – review & editing, Writing – original draft, Visualization, Validation, Software, Project administration, Methodology, Investigation, Formal analysis, Data curation, Conceptualization. **Johann Brand:** Writing – review & editing, Methodology. **Lucas Pieper:** Writing – review & editing, Methodology. **Florian Putz:** Writing – review & editing, Supervision. **Johanna Lott:** Writing – review & editing, Conceptualization. **Christoph Dickmann:** Writing – review & editing, Conceptualization. **Rainer Fietkau:** Writing – review & editing, Resources. **Stefanie Corradini:** Writing – review & editing, Resources. **Christoph Bert:** Writing – review & editing, Supervision, Methodology, Conceptualization. **Juliane Szkitsak:** Writing – review & editing, Validation, Supervision, Methodology, Conceptualization.

## Declaration of competing interest

The authors declare the following financial interests/personal relationships which may be considered as potential competing interests: B.-N.A., J.L. and C.D. are employees of Siemens Healthineers AG. The University Hospital Erlangen and the Department of Radiation Oncology have institutional research grants with Siemens Healthineers AG that are not directly related to the present work.

## Appendix A Supplementary data

Supplementary material Supplementary data to this article can be found online: S1: Comprehensive overview of all fourteen head phantoms evaluated for eligibility, including excluded candidates and inclusion rationale. S2: Bone, soft-tissue, and total-specimen segmentations on the reference CT, 2D and 3D synthetic CT for all included phantoms. S3: Representative axial RayStation screenshots illustrating dose distribution on reference CT and synthetic CT for selected phantoms. S4: Literature-derived acceptance thresholds for geometric, CT number, and dose metrics underlying the three-tier classification. S5: Alternative traffic-light thresholds for conventional fractionation (3% and 5% dose deviation variants).
