# Supplementary material for: Phantom benchmarking for quality assurance of synthetic computed tomography in cranial magnetic resonance-only radiotherapy
Source: Phys Imaging Radiat Oncol. 2026 Aug 14;40:101062. doi: 10.1016/j.phro.2026.101062 (PMC13544310; doi:10.1016/j.phro.2026.101062)
Supplement: Supplementary file 1 [file mmc1.pdf]

Supplementary material S1: Comprehensive phantom overview

Overview of all fourteen head phantoms evaluated for study inclusion, showing imaging data (CT, MR, 2D sCT, 3D sCT), material composition, and inclusion rationale. Ten phantoms were included in the final analysis (A–J); five were excluded due to limited stability, size incompatibility, or missing imaging data.

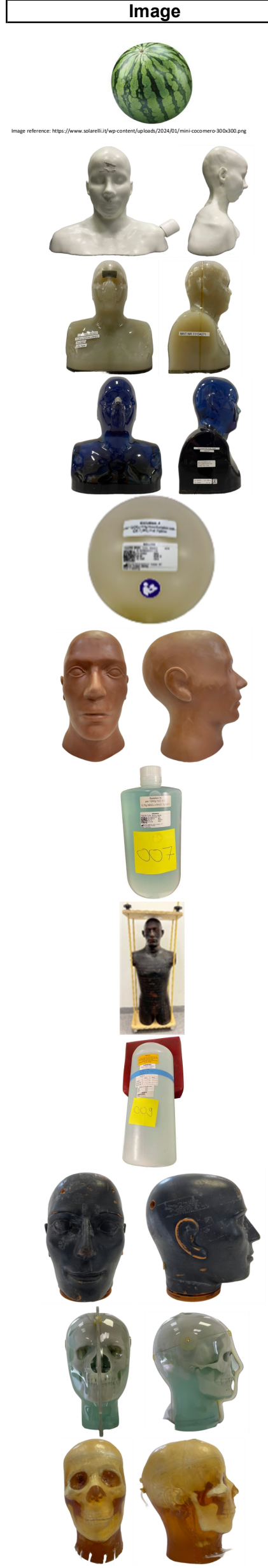

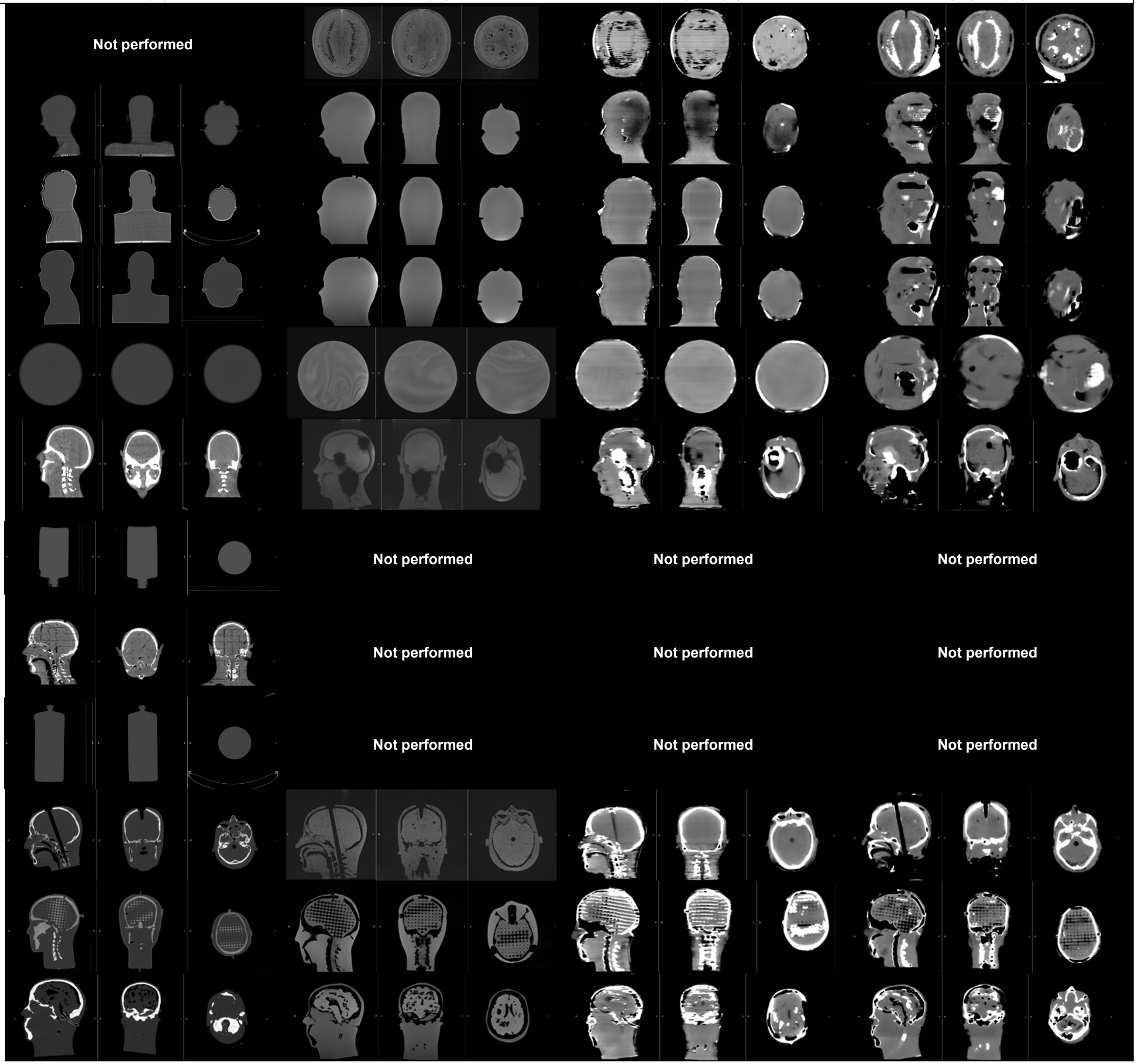

Image	Nr	Name	Material	Included	CT Done	MR Done	2D sCT Done	3D sCT Done
	1	Watermelon	Heterogeneous water-based organic medium; cellulose-based outer shell; lipid/protein-dense inclusions	No, limited stability	Not performed			
	2	MR HEAD WHITE	Proprietary homogeneous synthetic medium	Yes				
	3	MR HEAD AGAR	Homogeneous aqueous agar-based gel	Yes				
	4	MR HEAD BLUE	Homogeneous mineral oil-based medium	Yes				
	5	Spher. Phantom D165 Yellow	Homogeneous silicone oil-based medium	Yes				
	6	ExacTrac Dynamic Cranial Verification Phantom (Original, with tungsten-carbide spheres)	Bone-equivalent skull & cervical vertebrae; tissue-equivalent outer material; tungsten-carbide spheres	Yes				
	7	Plastic Bottle 1900ml	Homogeneous aqueous medium doped with paramagnetic ions and conductivity modifiers	No, non-anthropomorphic geometry		Not performed	Not performed	Not performed
	8	Alderson Full Body Phantom (ART/RANDO)	Proprietary epoxy-based soft-tissue equivalent (per ICRU-44) with cast polymer bone substitute; 2.5 cm slices	No, insufficient MRI imageability		Not performed	Not performed	Not performed
	9	Plastic Bottle 7300ml	Homogeneous aqueous medium doped with paramagnetic ions and conductivity modifiers	No, non-anthropomorphic geometry		Not performed	Not performed	Not performed
	10	ALDERSON Phantom Patient	Tissue-equivalent soft-tissue; polymer bone substitute (per ICRU-44)	Yes				
	11	MR Distortion and Image Fusion Head Phantom (Model 603-GS)	Plastic-based trabecular bone substitute; proprietary water-based polymer soft-tissue	Yes				
	12	Adult Head (Dynamic)	Ultra-soft polyurethane-based brain parenchyma; 3-layer bone-like skull, fillable internal blood vessels	Yes				

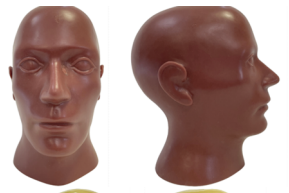	**13**	ExacTrac Dynamic Cranial Verification Phantom (Adapted, without tungsten-carbide spheres)	Bone-equivalent skull & cervical vertebrae; tissue-equivalent outer material	Yes	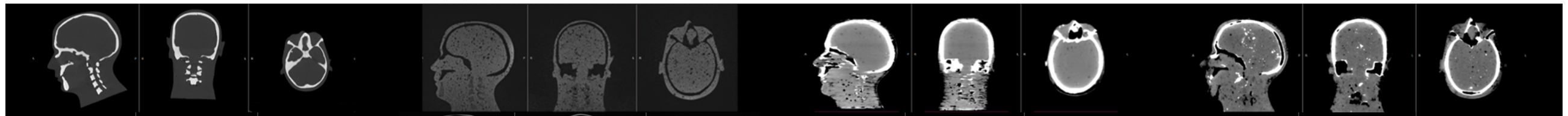
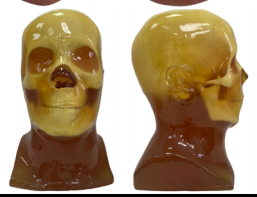	**14**	Adult Head (Static)	Urethane-based soft resin; ceramic-reinforced epoxy-based synthetic bone	Yes	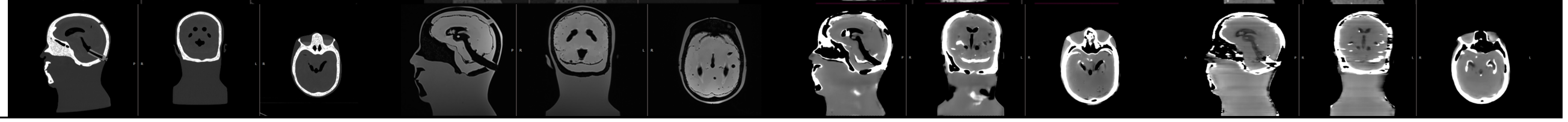

Supplementary Material S2: Visual phantom atlas

Axial CT slices and 3D segmentation renderings (total volume, bone, soft tissue) for each of the ten included phantoms (A–J), comparing rCT, 2D sCT, and 3D sCT. See main text for segmentation methodology.

Phantom A: Spherical Phantom D165, Siemens Healthineers AG

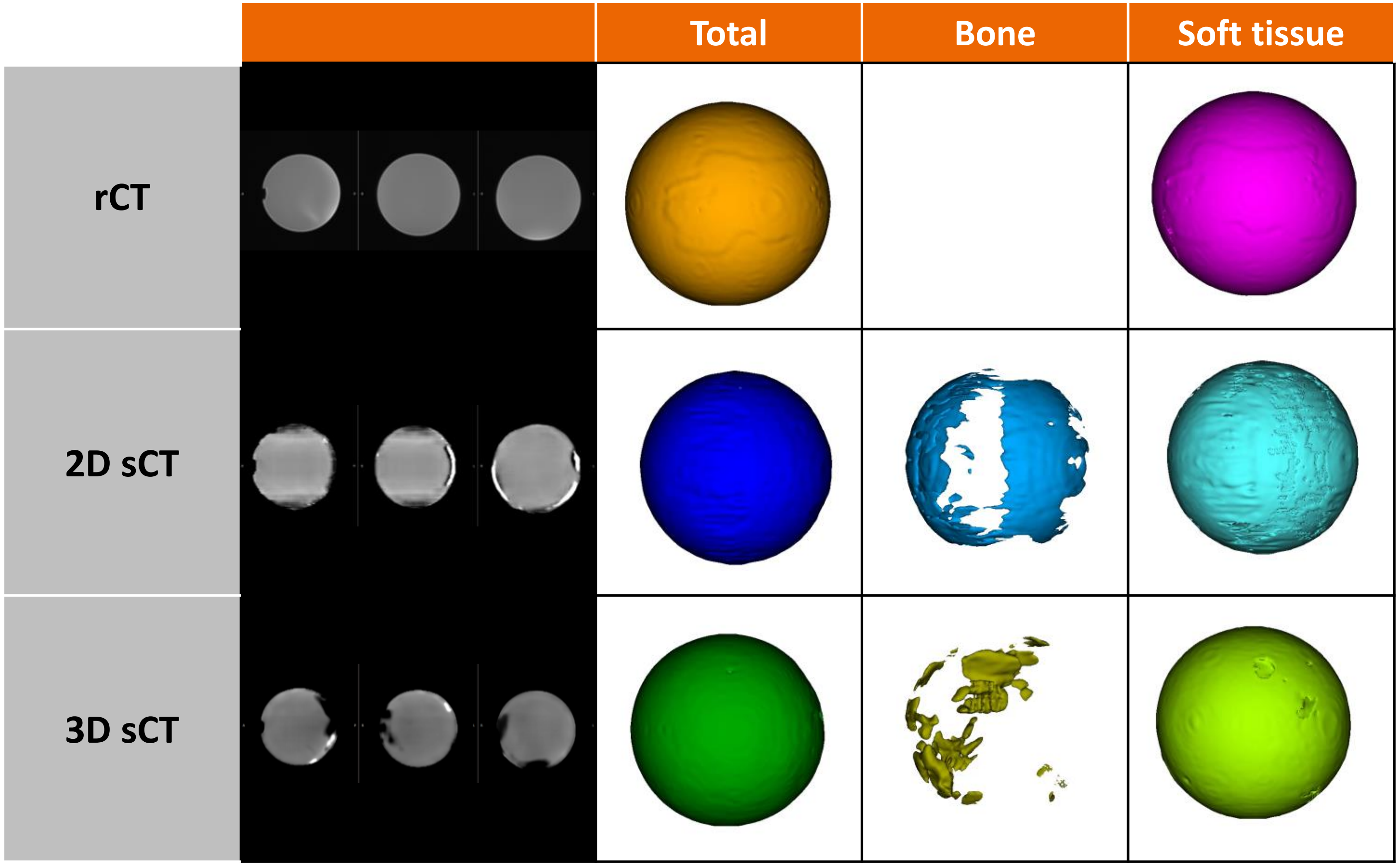

Axer BN, et al., Phantom benchmarking for quality assurance of synthetic computed tomography in cranial magnetic resonance-only radiotherapy. 2026

Phantom B: MR HEAD AGAR, Siemens Healthineers AG

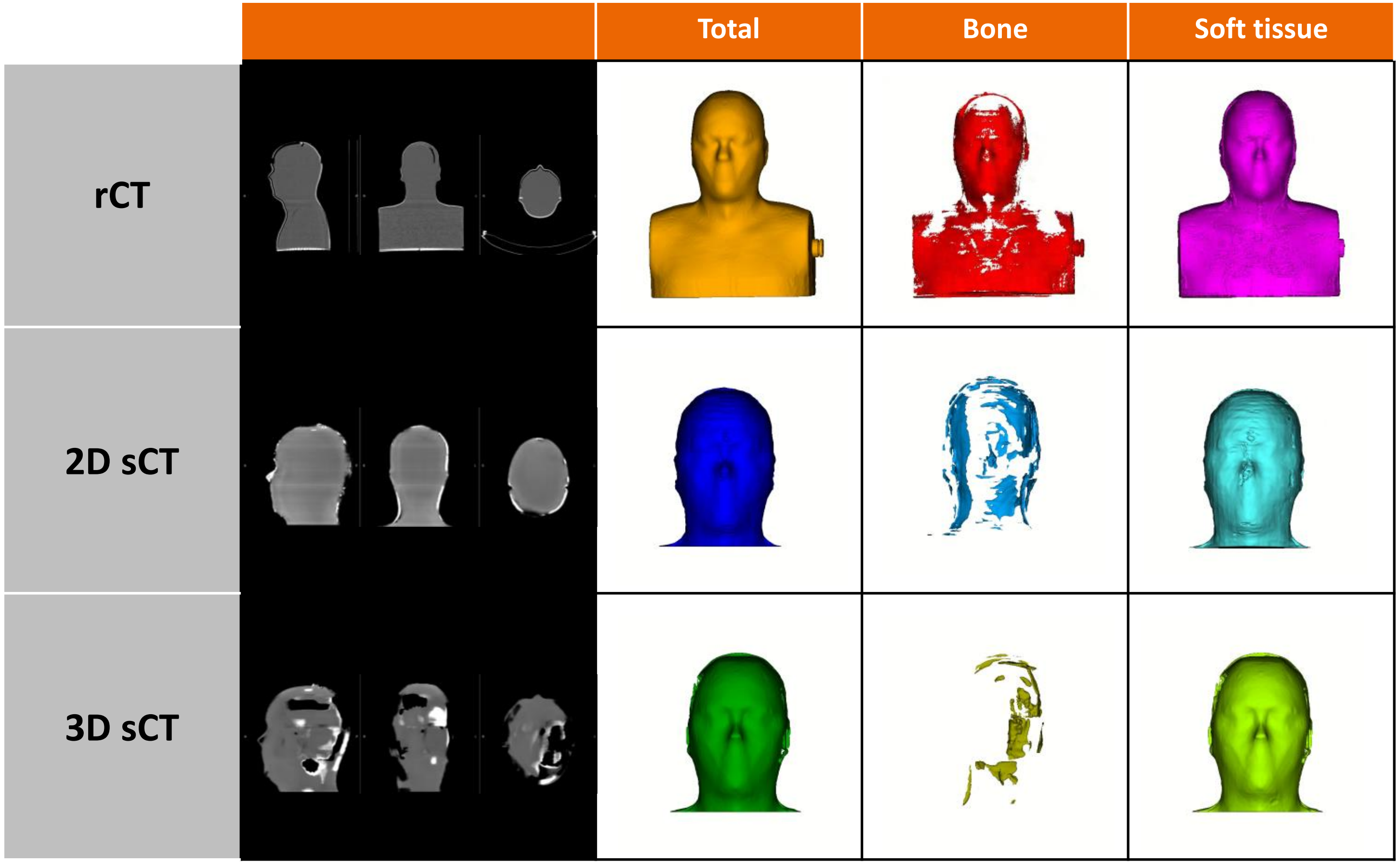

Phantom C: MR HEAD BLUE, Siemens Healthineers AG

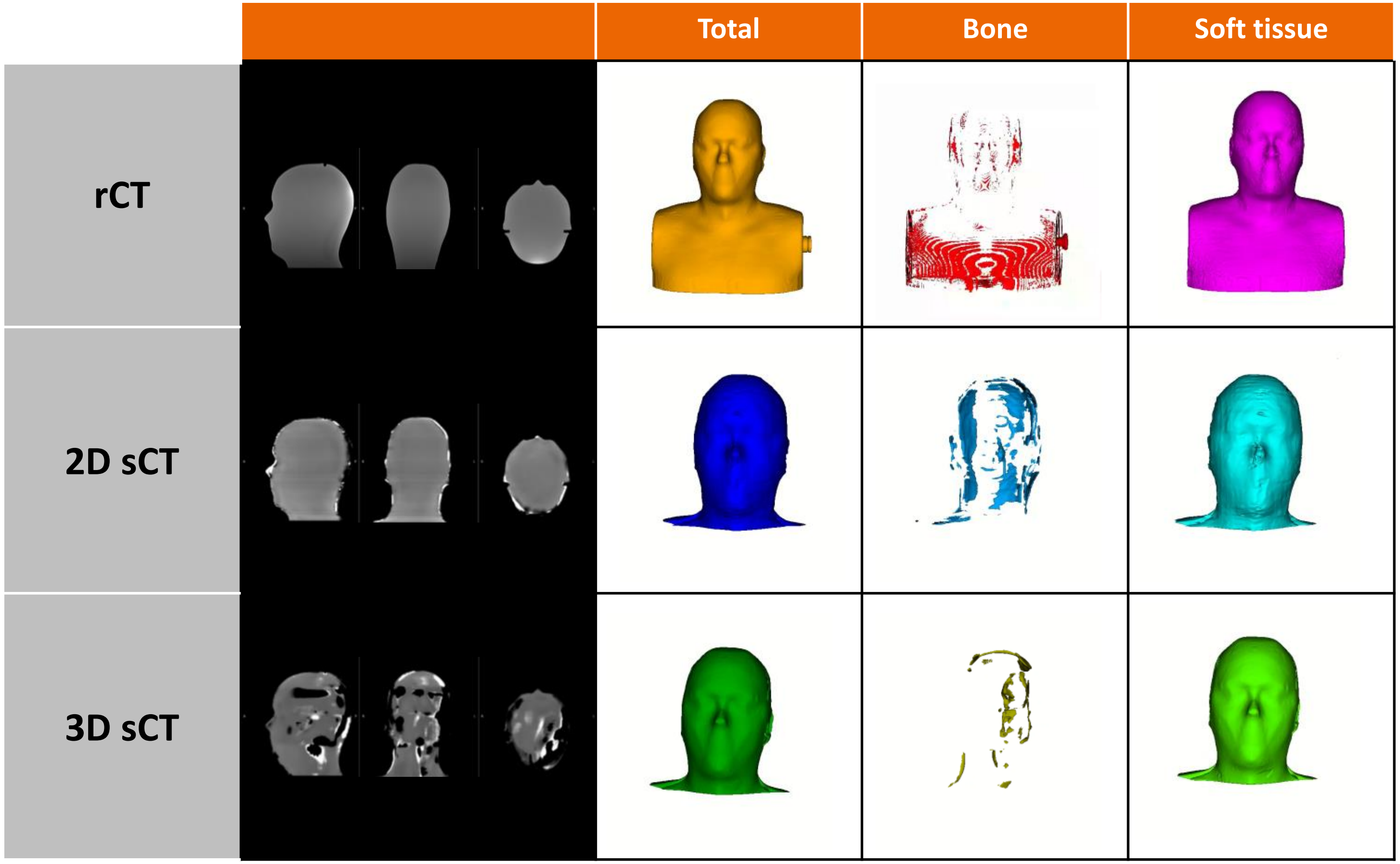

Phantom D: MR HEAD WHITE, Siemens Healthineers AG

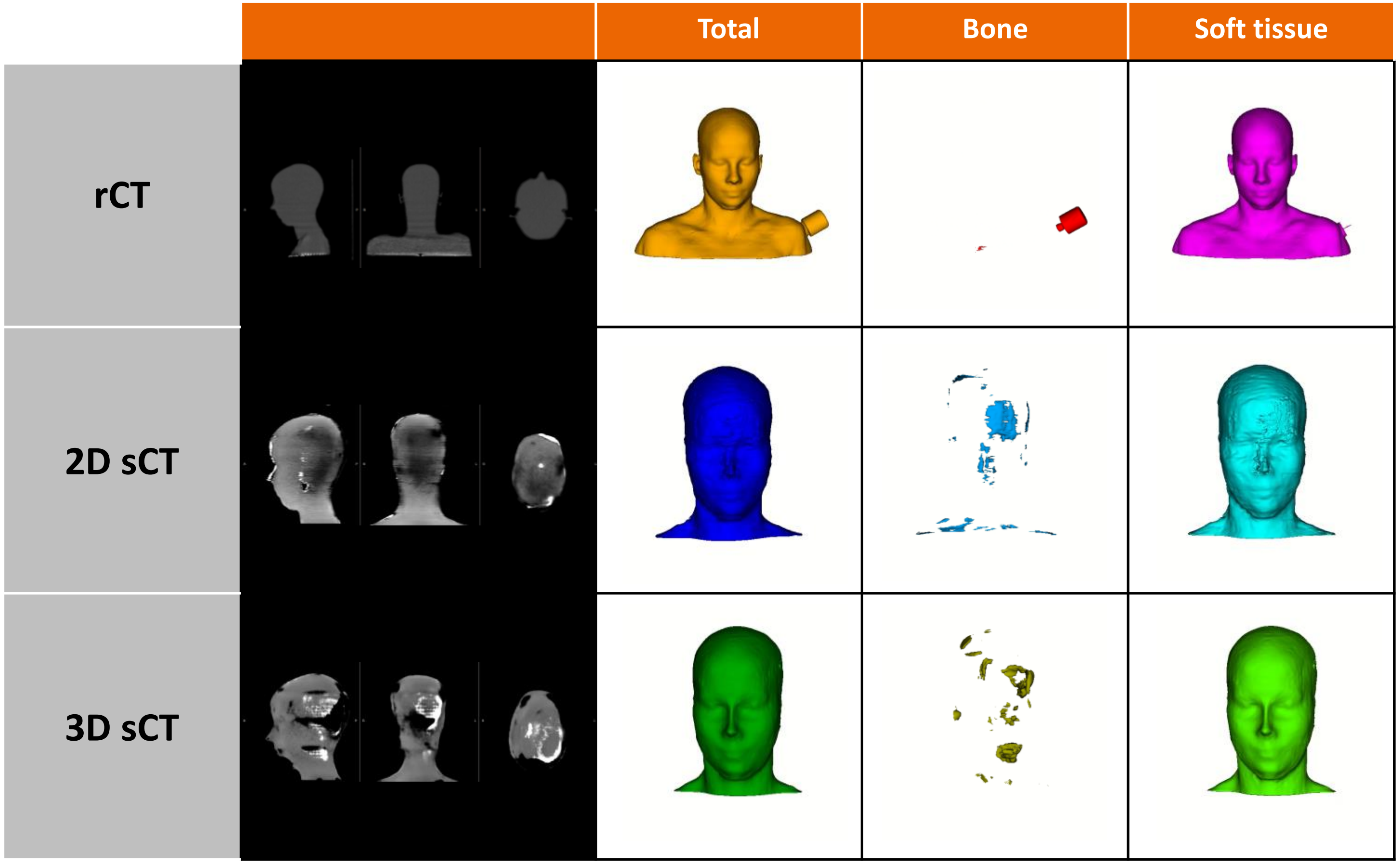

Axer BN, et al., Phantom benchmarking for quality assurance of synthetic computed tomography in cranial magnetic resonance-only radiotherapy. 2026

Phantom E: MR Distortion and Image Fusion Head Phantom (Model 603-GS), Sun Nuclear, a Mirion Medical Company

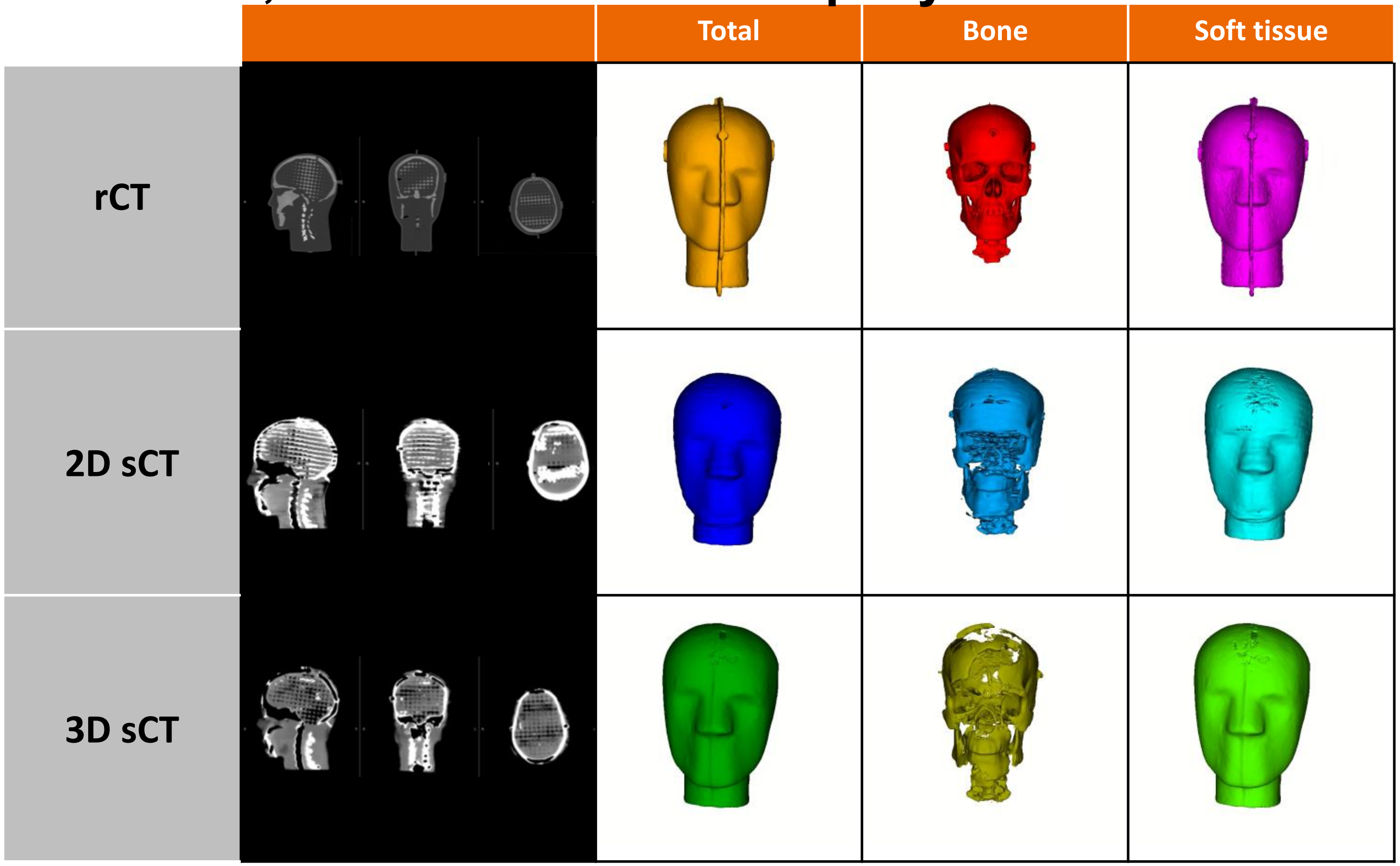

Axer BN, et al., Phantom benchmarking for quality assurance of synthetic computed tomography in cranial magnetic resonance-only radiotherapy. 2026

Phantom F: ALDERSON Phantom Patient, Radiology Support Devices

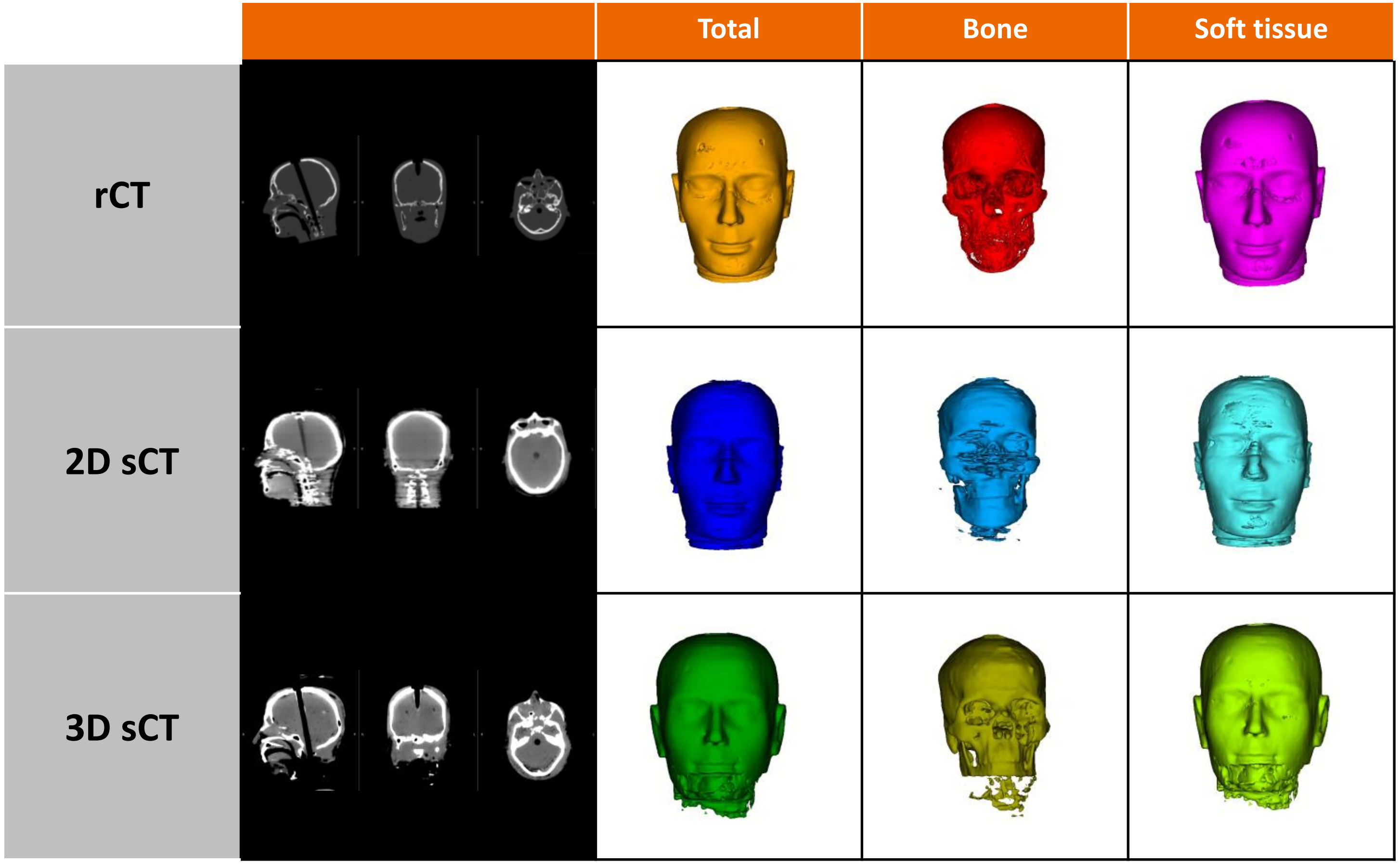

Axer BN, et al., Phantom benchmarking for quality assurance of synthetic computed tomography in cranial magnetic resonance-only radiotherapy. 2026

Phantom G: ExacTrac Dynamic Cranial Verification Phantom (Original), Brainlab SE

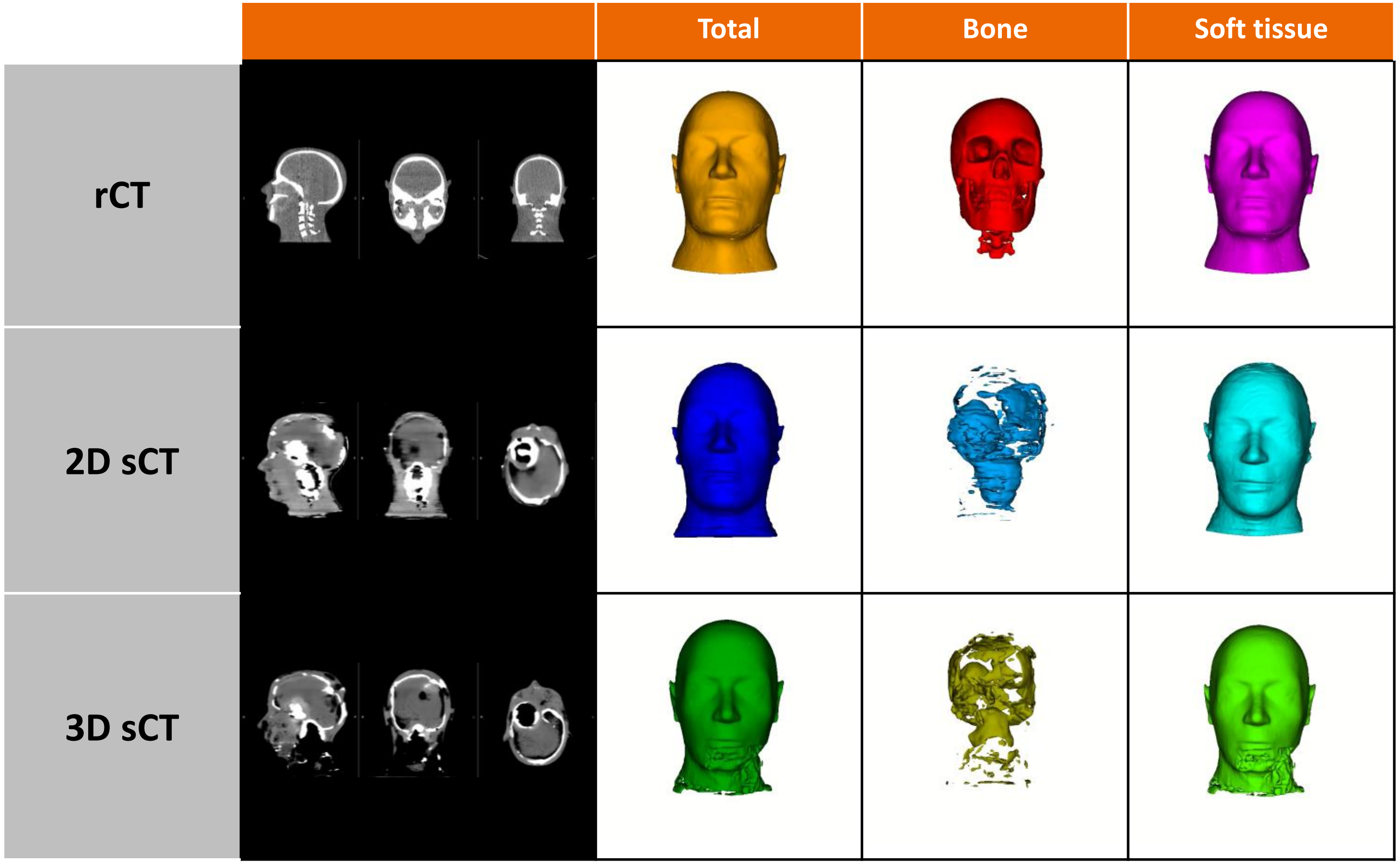

Phantom H: ExacTrac Dynamic Cranial Verification Phantom (Adapted), Brainlab SE

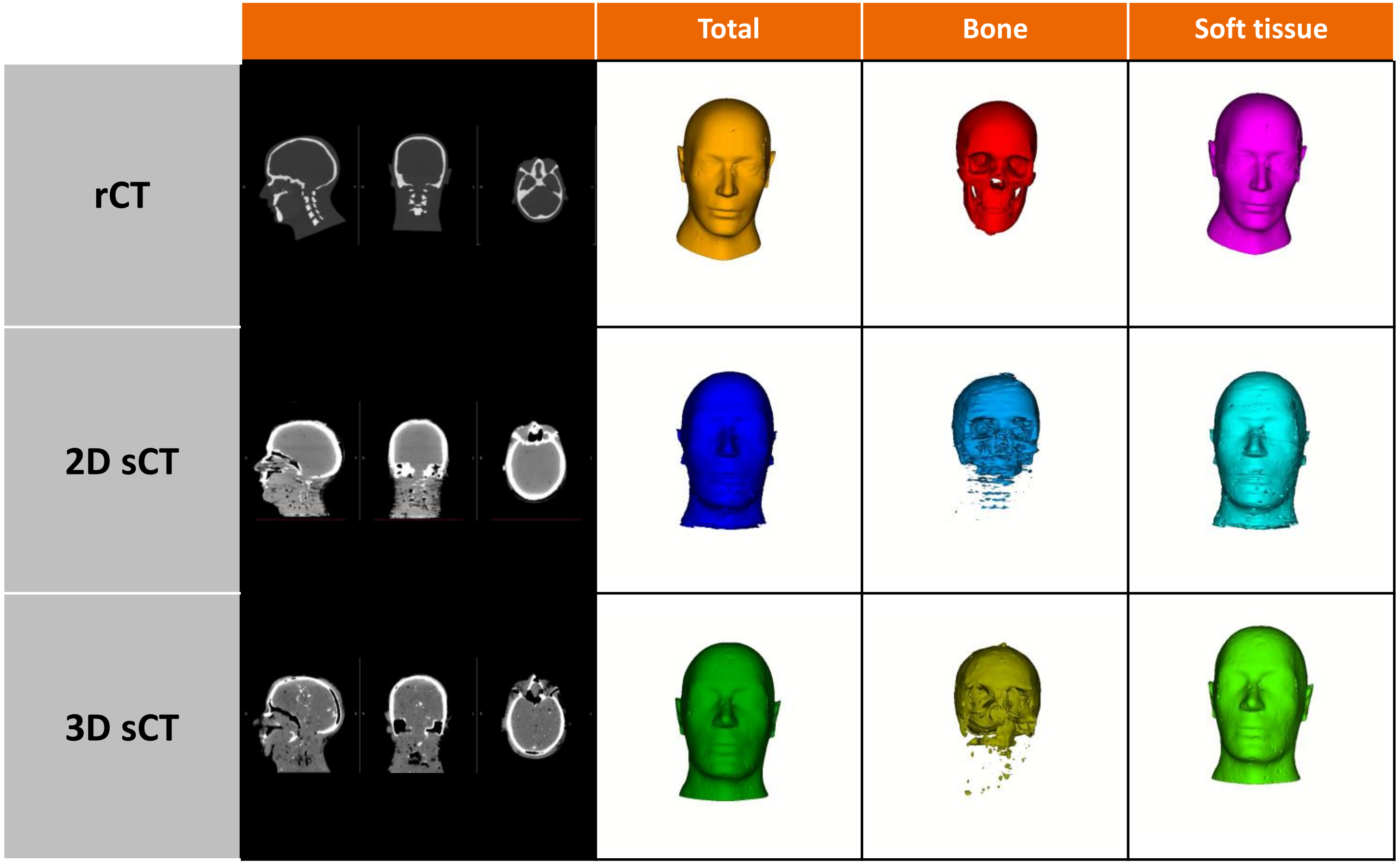

Axer BN, et al., Phantom benchmarking for quality assurance of synthetic computed tomography in cranial magnetic resonance-only radiotherapy. 2026

Phantom I: Adult Head (Dynamic), True Phantom Solutions

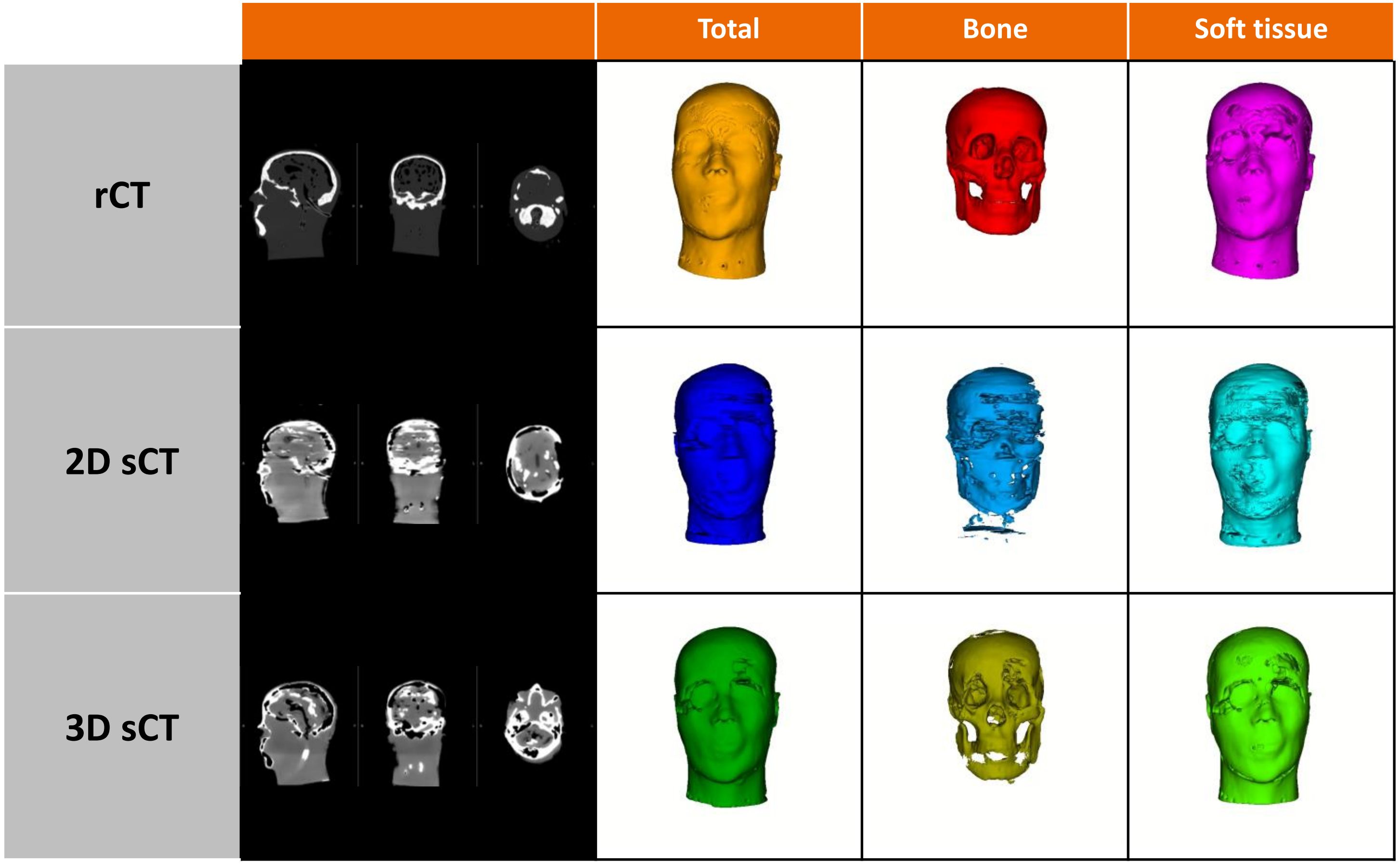

Axer BN, et al., Phantom benchmarking for quality assurance of synthetic computed tomography in cranial magnetic resonance-only radiotherapy. 2026

Phantom J: Adult Head (Static), True Phantom Solutions

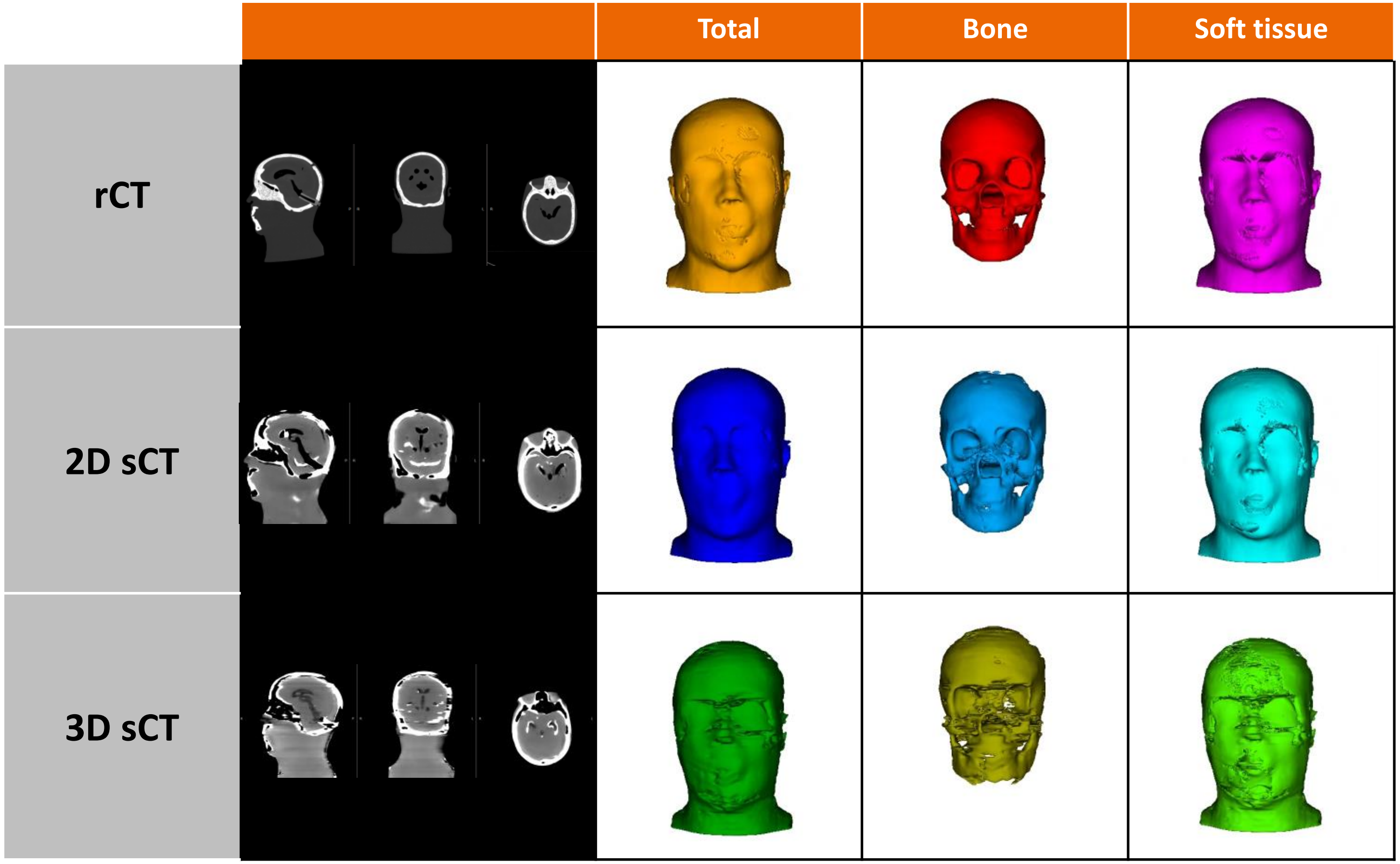

Axer BN, et al., Phantom benchmarking for quality assurance of synthetic computed tomography in cranial magnetic resonance-only radiotherapy. 2026

Supplementary material S3: Representative axial RayStation screenshots illustrating dose distribution on reference CT and synthetic CT for selected phantoms

Three of the ten head phantoms (A, D, and H) were selected for visual presentation to span the structural and performance range of the cohort: phantoms A and D represent the non-anthropomorphic group, with contrasting geometries (cylindrical and head-shaped without skull insert, respectively), while phantom H was included as the highest-ranked phantom in the composite quality assurance scoring reported in the main manuscript. Abbreviations follow the conventions defined in the main manuscript and are restated below for self-containment of this supplement.

For each of the three selected phantoms, dose distributions and voxel-wise dose-difference maps were exported from RayStation (version 12A, RaySearch Laboratories, Stockholm, Sweden) under identical window/level and isodose settings to ensure visual comparability. Each figure follows the same matrix layout: rows correspond to the rCT, the 2D sCT, the 3D sCT, and the voxel-wise dose differences rCT − 2D sCT and rCT − 3D sCT; columns show the VMAT plan and the APLR four-field plan, each in transversal, coronal, and sagittal view.

Abbreviations: rCT, reference computed tomography; sCT, synthetic computed tomography; VMAT, volumetric modulated arc therapy; APLR, anterior–posterior / left–right four-field plan.

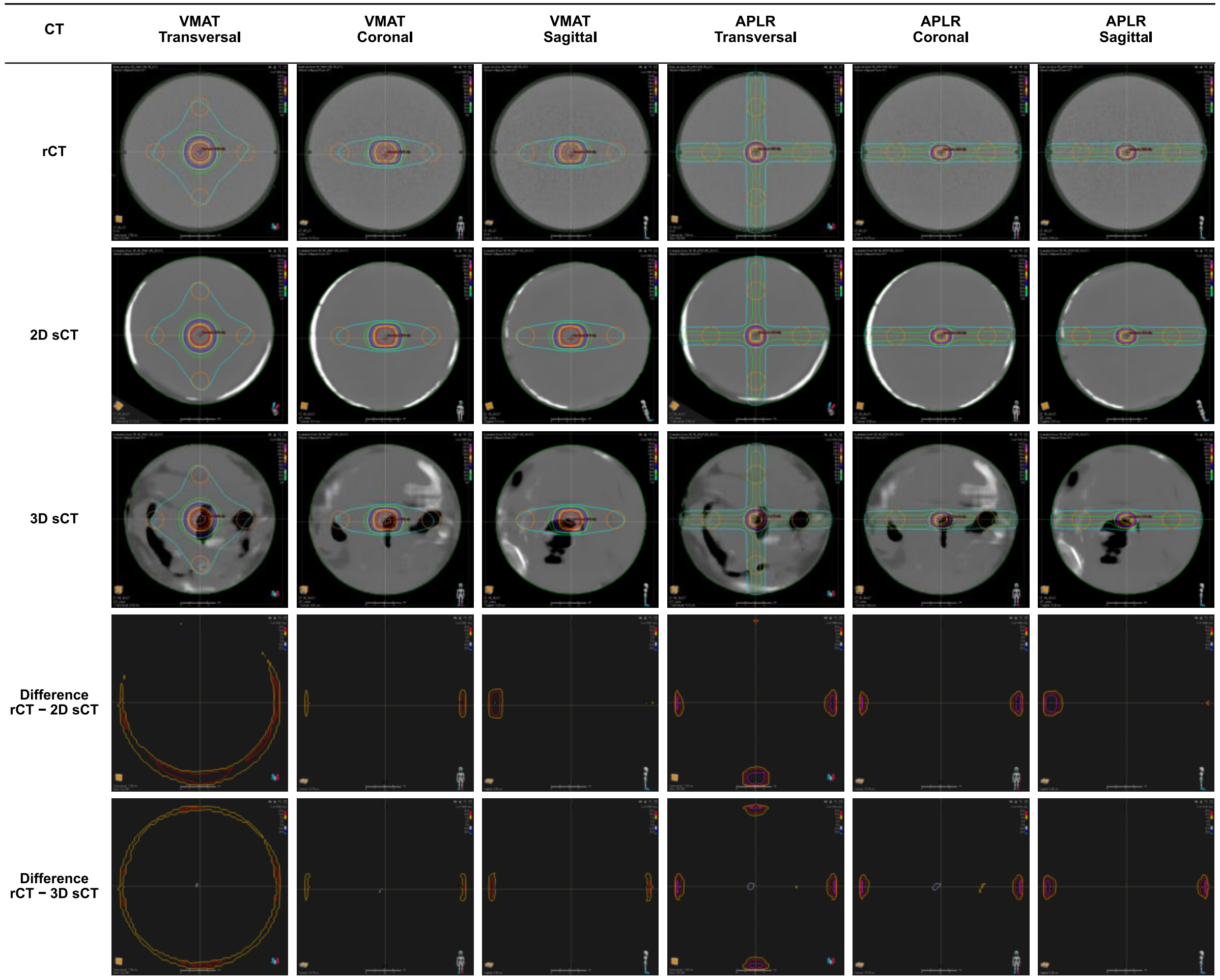

Figure 3.1 Dose distributions and dose-difference maps for phantom A. Layout as described.

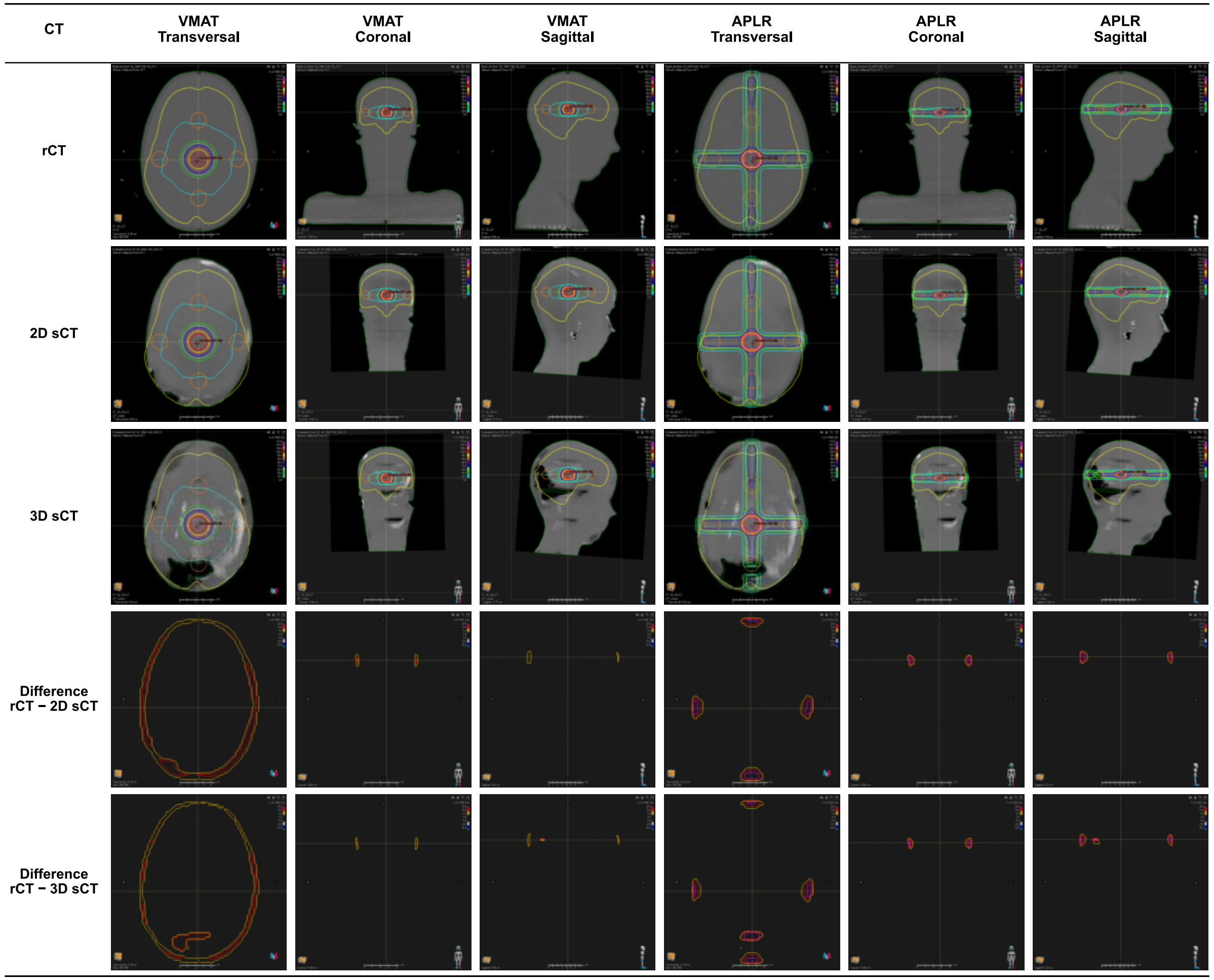

Figure 3.2 Dose distributions and dose-difference maps for phantom D. Layout as described.

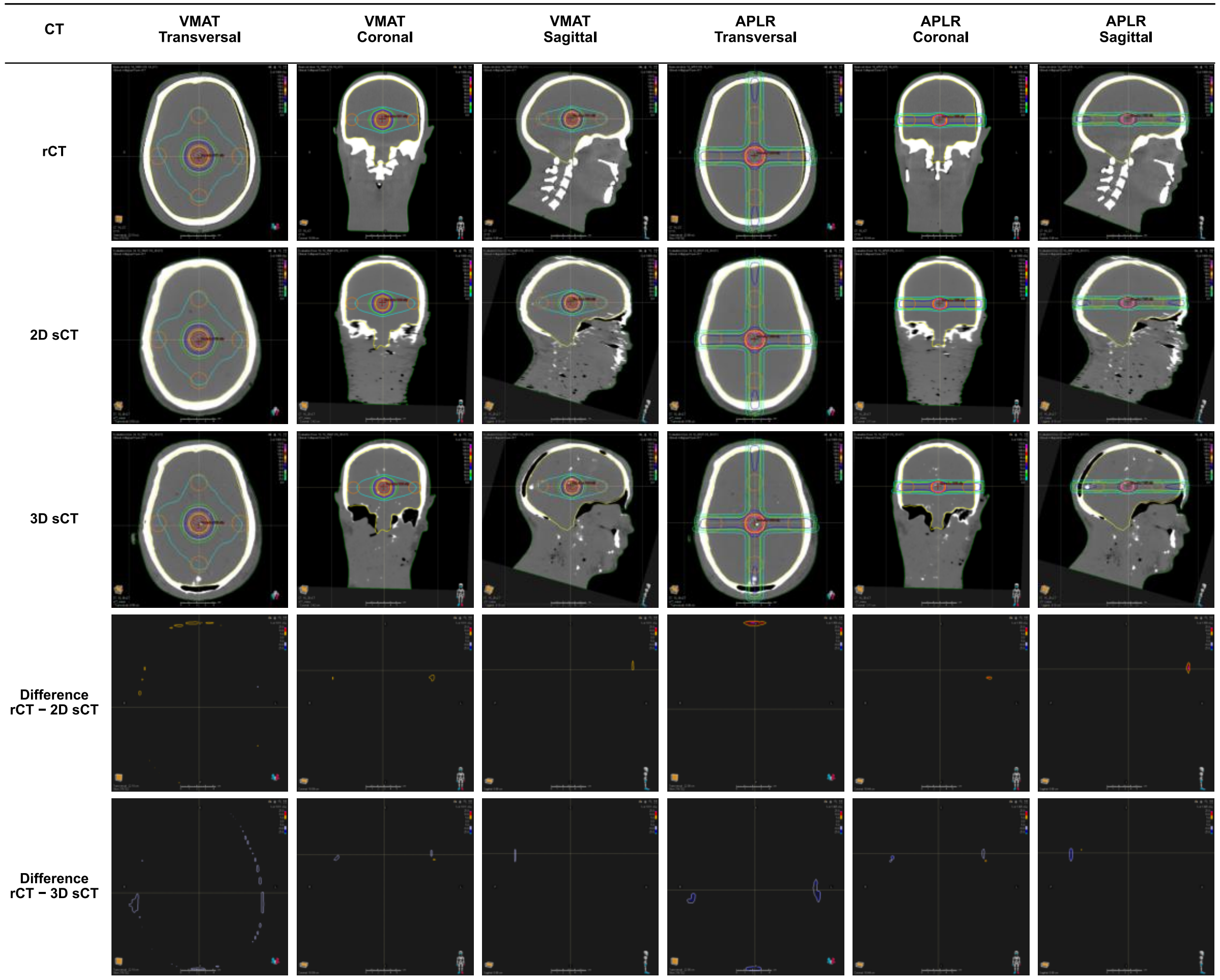

Figure 3.3 Dose distributions and dose-difference maps for phantom D. Layout as described.

Supplementary material S4: Literature-derived acceptance thresholds

Literature-derived acceptance thresholds for geometric, CT number, and dose metrics underlying the three-tier classification.

Dice Similarity Coefficient (DSC) Bone Segmentation

Tier	DSC Threshold	Study	Year	Type	DSC bone	Ref.
Green	≥ 0.8	Brock et al.	2017	Guideline	0.80 – 0.90	[1]
Green	≥ 0.8	Spadea et al.	2021	Patient	0.93 ± 0.02	[2]
Green	≥ 0.8	Lerner et al.	2021	Patient	0.92 ± 0.01	[3]
Green	≥ 0.8	Maspero et al.	2020	Patient	0.92 ± 0.05	[4]
Green	≥ 0.8	Earwong et al.	2025	Patient	0.87 ± 0.03	[5]
Yellow	0.6 ≤ DSC < 0.8	Palmér et al.	2021	Patient	0.80 ± 0.07	[6]
Yellow	0.6 ≤ DSC < 0.8	Kazemifar et al.	2019	Patient	0.80 ± 0.06	[7]
Yellow	0.6 ≤ DSC < 0.8	Dinkla et al.	2019	Patient	0.70 ± 0.07	[8]
Yellow	0.6 ≤ DSC < 0.8	Olin et al.	2021	Patient	0.67 ± 0.05	[9]
Red	< 0.6	Below reported and accepted cranial bone DSC values				

Hausdorff Distance 95th (HD95) Bone Segmentation

Tier	HD95 Threshold (mm)	Study	Year	Type	HD95 / Geometric accuracy (mm)	Ref.
Green	≤ 2.0	Schmitt et al.	2020	Guideline	≤ 1.25	[10]
Green	≤ 2.0	Halvorsen et al.	2017	Guideline	≤ 2.0	[11]
Green	≤ 2.0	Kuncman et al.	2025	Review	≤ 2.0	[12]
Yellow	2.0 < HD95 ≤ 5.0	Palmér et al.	2021	Patient	4.6 ± 1.2	[6]
Red	> 5.0	Above reported and accepted cranial bone HD values				

Mean Error (ME) CT Number Bone Segmentation

Tier	|ME| Threshold (HU)	Study	Year	Type	ME (HU)	Ref.
Green	≤ 100	Lerner et al.	2021	Patient	-41.9 ± 18.3	[3]
Green	100 < |ME| ≤ 400	Palmér et al.	2021	Patient	-62 ± 28	[6]
Yellow	100 < |ME| ≤ 400	Dinkla et al.	2019	Patient	106 ± 51	[8]
Yellow	100 < |ME| ≤ 400	Olin et al.	2021	Patient	-199 ± 60	[9]
Yellow	100 < |ME| ≤ 400	Autret et al.	2023	Patient	-381	[13]
Red	> 400	Above reported and accepted cranial bone HU ME values				

Mean Error (ME) CT Number Soft Tissue Segmentation

Tier	|ME| Threshold (HU)	Study	Year	Type	ME (HU)	Ref.
Green	≤ 10	Palmér et al.	2021	Patient	-1 ± 7	[6]
Green	10 < |ME| ≤ 20	Dinkla et al.	2019	Patient	-2 ± 5	[8]
Yellow	10 < |ME| ≤ 20	Olin et al.	2021	Patient	-9 ± 6	[9]
Red	> 20	Above reported and accepted cranial bone HU ME values				

Dose deviations (ΔD_x)

Tier	$\lvert\Delta D_x\rvert$ Threshold (%)	Study	Year	Type	Dose deviation (%) with Standard Deviation (±) or range (-)	Ref.
Green	≤ 1.0	Maspero et al.	2020	Patient	$\Delta D_{90\%}$ = -0.1 ± 0.3<br>$\Delta D_{10\%}$ = -0.0 ± 0.3<br>$\Delta D_{2\%}$, $\Delta D_{50\%}$, ΔD_{max} < 0.5	[4]
Green	≤ 1.0	Paradis et al.	2015	Patient	$\Delta D_{95\%}$ = 0.0 (-0.1 – 0.2)<br>$\Delta D_{5\%}$ = 0.0 (-0.7 – 0.6)<br>ΔD_{max} = -0.2 (-1.0 – 0.2)	[14]
Green	≤ 1.0	Dinkla et al.	2017	Patient	$\Delta D_{50\%}$ = 0.12 ± 0.28<br>$\Delta D_{10\%}$ = 0.32 ± 0.25<br>$\Delta D_{90\%}$ = -0.07 ± 0.22	[8]
Green	≤ 1.0	Lerner et al.	2021	Patient	ΔD_{mean} = 0.1 ± 0.2<br>$\Delta D_{98\%}$ = 0.1 ± 0.2<br>$\Delta D_{2\%}$ = 0.0 ± 0.2	[3]
Green	≤ 1.0	Palmér et al.	2021	Patient	ΔD_{mean} = -0.1 ± 0.2<br>$\Delta D_{98\%}$ = -0.0 ± 0.4<br>$\Delta D_{2\%}$ = 0.0 ± 0.4<br>$\Delta D_{98\%}$ = -0.1 ± 0.2	[6]
Green	≤ 1.0	Aljaafari et al.	2025	Patient	$\Delta D_{50\%}$ = 0.3 (0.2 – 0.3)<br>$\Delta D_{2\%}$ = 0.4 (0.3 – 0.5)<br>$\Delta D_{98\%}$ = 0.2 (0.1 – 0.2)	[15]

Tier	$\lvert\Delta D_x\rvert$ Threshold (%)	Study	Year	Type	Dose deviation (%) with Standard Deviation (±) or range (-)	Ref.
Green	≤ 1.0	Kazemifar et al.	2019	Patient	ΔD_{mean} = -0.7 (-1.8 – 1.2)<br>ΔD_{max} = -0.6 (-2.3 – 0.3)<br>$\Delta D_{95\%}$ = -0.6 (-2.2 – 0.3)<br>$\Delta D_{5\%}$ = -0.7 (-2.2 – 0.4)	[7]
Yellow	1.0 < $\lvert\Delta Dx\rvert$ ≤ 2.0	Farjam et al.	2017	Patient	ΔD_{max} = 0.34 ± 0.38<br>ΔD_{mean} = 0.38 ± 0.4<br>$\Delta D_{95\%}$ = 1.12 ± 2.1	[16]
Yellow	1.0 < $\lvert\Delta Dx\rvert$ ≤ 2.0	Korsholm et al.	2014	Patient	ΔD_{median} = -0.6 ± 1.2<br>$\Delta D_{98\%}$ = -0.006 ± 1.8<br>$\Delta D_{98\%}$ = -0.4 ± 0.9	[17]
Red	> 2.0	Olin et al.	2023	Patient	$\Delta D_{2\%}$, $\Delta D_{50\%}$, $\Delta D_{98\%}$, ΔD_{mean}, ΔD_{max}, ΔD_{1cc}, $\Delta D_{0.1cc}$ < 2.3	[9]
Red	> 2.0	Masitho et al.	2023	Patient	$\Delta D_{50\%}$ ≤ 2.0	[18]

References

[1] Brock KK, Mutic S, McNutt TR, Li H, Kessler ML. Use of image registration and fusion algorithms and techniques in radiotherapy: Report of the AAPM Radiation Therapy Committee Task Group No. 132. Med Phys 2017;44:e43–76. https://doi.org/10.1002/mp.12256.

[2] Spadea MF, Maspero M, Zaffino P, Seco J. Deep learning based synthetic-CT generation in radiotherapy and PET: A review. Med Phys 2021;48:6537–66. https://doi.org/10.1002/mp.15150.

[3] Lerner M, Medin J, Jamtheim Gustafsson C, Alkner S, Siversson C, Olsson LE. Clinical validation of a commercially available deep learning software for synthetic CT generation for brain. Radiat Oncol 2021;16:66. https://doi.org/10.1186/s13014-021-01794-6.

[4] Maspero M, Bentvelzen LG, Savenije MHF, Guerreiro F, Seravalli E, Janssens GO, et al. Deep learning-based synthetic CT generation for paediatric brain MR-only photon and proton radiotherapy. Phys Spec Issue ESTRO Phys Res Workshop Sci Dev 2020;153:197–204. https://doi.org/10.1016/j.radonc.2020.09.029.

[5] Earwong P, Puttanawarut C, Suphaphong S, Worapruekjaru L, Jiarpinitnun C, Sawapabmongkon T, et al. Clinical implementation of deep learning-based synthetic CT for MRI-only volumetric modulated arc therapy in head and neck and pelvic cancer patients. Radiat Oncol Lond Engl 2025;20:166. https://doi.org/10.1186/s13014-025-02744-2.

[6] Palmér E, Karlsson A, Nordström F, Petruson K, Siversson C, Ljungberg M, et al. Synthetic computed tomography data allows for accurate absorbed dose calculations in a magnetic resonance imaging only workflow for head and neck radiotherapy. Phys Imaging Radiat Oncol 2021;17:36–42. https://doi.org/10.1016/j.phro.2020.12.007.

[7] Kazemifar S, McGuire S, Timmerman R, Wardak Z, Nguyen D, Park Y, et al. MRI-only brain radiotherapy: Assessing the dosimetric accuracy of synthetic CT images generated using a deep learning approach. Radiother Oncol 2019;136:56–63. https://doi.org/10.1016/j.radonc.2019.03.026.

[8] Dinkla AM, Florkow MC, Maspero M, Savenije MHF, Zijlstra F, Doornaert PAH, et al. Dosimetric evaluation of synthetic CT for head and neck radiotherapy generated by a patch-based three-dimensional convolutional neural network. Med Phys 2019;46:4095–104. https://doi.org/10.1002/mp.13663.

[9] Olin AB, Thomas C, Hansen AE, Rasmussen JH, Krokos G, Urbano TG, et al. Robustness and Generalizability of Deep Learning Synthetic Computed Tomography for Positron Emission Tomography/Magnetic Resonance Imaging–Based Radiation Therapy Planning of Patients With Head and Neck Cancer. Adv Radiat Oncol 2021;6. https://doi.org/10.1016/j.adro.2021.100762.

[10] Schmitt D, Blanck O, Gauer T, Fix MK, Brunner TB, Fleckenstein J, et al. Technological quality requirements for stereotactic radiotherapy. Strahlenther Onkol 2020;196:421–43. https://doi.org/10.1007/s00066-020-01583-2.

[11] Halvorsen PH, Cirino E, Das IJ, Garrett JA, Yang J, Yin F-F, et al. AAPM-RSS Medical Physics Practice Guideline 9.a. for SRS-SBRT. J Appl Clin Med Phys 2017;18:10–21. https://doi.org/10.1002/acm2.12146.

[12] Kuncman Ł, la Pinta CD, Milder MTW, Romero AM, Guckenberger M, Franzese C, et al. Definition and requirements for stereotactic radiotherapy: a systematic review. Radiother Oncol 2025;211. https://doi.org/10.1016/j.radonc.2025.111107.

[13] Autret D, Guillerminet C, Roussel A, Cossec-Kerloc'h E, Dufreneix S. Comparison of four synthetic CT generators for brain and prostate MR-only workflow in radiotherapy. Radiat Oncol 2023;18:146. https://doi.org/10.1186/s13014-023-02336-y.
[14] Paradis E, Cao Y, Lawrence TS, Tsien C, Feng M, Vineberg K, et al. Assessing the dosimetric accuracy of magnetic resonance-generated synthetic CT images for focal brain VMAT radiation therapy. Int J Radiat Oncol Biol Phys 2015;93:1154–61. https://doi.org/10.1016/j.ijrobp.2015.08.049.
[15] Aljaafari L, Speight R, Buckley DL, Bird D, Al-Qaisieh B. Clinical validation of using a commercial synthetic-computed tomography solution for brain MRI-only radiotherapy treatment planning. Tech Innov Patient Support Radiat Oncol 2025;35:100328. https://doi.org/10.1016/j.tipsro.2025.100328.
[16] Farjam R, Tyagi N, Veeraraghavan H, Apte A, Zakian K, Hunt MA, et al. Multiatlas approach with local registration goodness weighting for MRI-based electron density mapping of head and neck anatomy. Med Phys 2017;44:3706–17. https://doi.org/10.1002/mp.12303.
[17] Korsholm ME, Waring LW, Edmund JM. A criterion for the reliable use of MRI-only radiotherapy. Radiat Oncol 2014;9:16. https://doi.org/10.1186/1748-717X-9-16.
[18] Masitho S, Grigo J, Brandt T, Lambrecht U, Szkitsak J, Weiss A, et al. Synthetic CTs for MRI-only brain RT treatment: integration of immobilization systems. Strahlenther Onkol 2023;199:739–48. https://doi.org/10.1007/s00066-023-02090-w.

Supplementary material S5: Dosimetric heatmaps with alternative acceptance thresholds and additional DVH metrics

Traffic-light heatmaps of relative PTV dose deviations (%) for the 2D and 3D sCT algorithm across six DVH metrics (D0.01cc, D2%, D50%, Dmean, D98%, D99%) and six beam configurations (VMAT, APLR, AP, PA, LR, RL). Each figure shows a different threshold combination. Fig. 4 in the main text shows D2%, Dmean, and D98% with stereotactic thresholds (green ≤ 1%, red > 2%).

a) 2D synthetic vs. reference CT: PTV Dose Deviations (Green ≤1.0%, Yellow ≤2.0%, Red >2.0%)

VMAT

	$\Delta D_{0.01cc}$	$\Delta D_{2\%}$	$\Delta D_{50\%}$	ΔD_{mean}	$\Delta D_{98\%}$	$\Delta D_{99\%}$
A	+3.9	+3.9	+3.8	+3.9	+3.9	+3.8
B	+2.5	+2.3	+2.2	+2.2	+2.2	+2.0
C	-3.2	-3.2	-3.0	-2.9	-2.5	-2.2
D	+3.3	+3.2	+3.3	+3.3	+3.2	+3.2
E	+2.5	+2.5	+2.0	+2.0	+1.5	+1.5
F	+4.6	+4.7	+1.7	+2.1	+1.5	+1.6
G	+1.2	+1.3	+1.1	+1.0	+0.9	+0.8
H	-0.2	-0.2	-0.2	-0.2	-0.2	-0.3
I	+3.3	+3.4	+2.7	+2.5	+1.0	+1.5
J	+0.7	+0.8	+1.1	+1.1	+1.8	+2.5

APLR

	$\Delta D_{0.01cc}$	$\Delta D_{2\%}$	$\Delta D_{50\%}$	ΔD_{mean}	$\Delta D_{98\%}$	$\Delta D_{99\%}$
A	+3.8	+3.7	+3.5	+3.6	+3.6	+3.6
B	+3.5	+3.2	+3.3	+2.9	+2.6	+2.7
C	-2.8	-2.7	-2.1	-2.3	-2.0	-2.8
D	+3.1	+3.2	+3.1	+3.1	+3.0	+3.2
E	+3.9	+3.8	+3.6	+3.5	+2.8	+1.7
F	+2.7	+3.5	+8.5	+6.5	+12.8	+0.9
G	+1.2	+1.0	+1.1	+1.0	+0.7	+1.1
H	-0.5	-0.5	-0.6	-0.5	-0.3	-0.3
I	+5.0	+5.1	+6.8	+5.6	+4.5	+4.3
J	+3.7	+3.7	+4.6	+4.1	+7.5	+2.8

AP

	$\Delta D_{0.01cc}$	$\Delta D_{2\%}$	$\Delta D_{50\%}$	ΔD_{mean}	$\Delta D_{98\%}$	$\Delta D_{99\%}$
A	+1.1	+1.1	+1.1	+1.1	+1.0	+1.2
B	+6.4	+6.3	+6.3	+6.3	+6.3	+6.0
C	-3.1	-3.2	-3.3	-3.2	-3.2	-4.4
D	+1.6	+1.4	+1.6	+1.5	+1.7	+1.6
E	+1.6	+2.6	+2.0	+2.5	+1.2	+2.0
F	+3.6	+3.9	+8.7	+9.0	+2.3	+1.8
G	-1.3	-1.1	-3.5	-2.0	+2.6	-2.0
H	+0.3	+0.6	+0.2	+0.3	+0.4	+0.5
I	+11.9	+11.8	+8.8	+10.7	+6.5	+9.5
J	+9.5	+9.2	+9.2	+9.1	+5.7	+4.9

PA

	$\Delta D_{0.01cc}$	$\Delta D_{2\%}$	$\Delta D_{50\%}$	ΔD_{mean}	$\Delta D_{98\%}$	$\Delta D_{99\%}$
A	+6.7	+6.5	+6.8	+6.8	+6.9	+6.6
B	+0.3	+0.2	+0.3	+0.3	+0.2	+0.3
C	-2.3	-2.4	-2.7	-2.5	-3.0	-3.4
D	+4.5	+4.6	+4.7	+4.7	+4.5	+4.4
E	+5.2	+6.0	+4.4	+4.7	+3.2	+11.7
F	-0.9	-0.5	+4.4	+3.2	-0.7	-0.3
G	+2.9	+2.6	+2.5	+2.3	-0.7	+2.2
H	-0.8	-1.0	-0.7	-0.8	-0.9	-0.7
I	+0.9	+1.0	+4.4	+3.6	+1.1	+1.5
J	+3.9	+3.9	+6.9	+6.1	+1.6	+3.6

LR

	$\Delta D_{0.01cc}$	$\Delta D_{2\%}$	$\Delta D_{50\%}$	ΔD_{mean}	$\Delta D_{98\%}$	$\Delta D_{99\%}$
A	+4.2	+4.1	+4.2	+4.1	+4.1	+4.1
B	+3.0	+3.1	+2.3	+2.4	+2.0	+0.9
C	-2.2	-2.2	-2.1	-2.3	-2.8	-2.5
D	+2.9	+3.0	+2.9	+3.0	+2.9	+3.0
E	+3.6	+3.5	+1.9	+2.2	+1.6	+1.3
F	+0.5	+0.6	+5.9	+6.4	+3.4	+2.4
G	+1.0	+1.2	+0.9	+1.1	+1.5	+1.5
H	-1.0	-1.2	-0.9	-1.0	-0.9	-0.9
I	+4.9	+5.4	+7.7	+7.7	+6.5	+5.6
J	-0.9	-0.9	+2.3	+1.1	+2.8	+2.8

RL

	$\Delta D_{0.01cc}$	$\Delta D_{2\%}$	$\Delta D_{50\%}$	ΔD_{mean}	$\Delta D_{98\%}$	$\Delta D_{99\%}$
A	+2.3	+2.5	+2.7	+2.4	+2.0	+2.2
B	+4.5	+3.1	+3.3	+3.4	+3.0	+2.0
C	-0.6	-1.2	-1.1	-1.0	-1.9	-1.7
D	+3.2	+3.0	+3.3	+3.2	+3.3	+3.2
E	+3.9	+4.3	+5.1	+4.4	+3.9	+3.6
F	+0.1	+0.1	+7.2	+7.5	+9.8	+2.6
G	+2.5	+2.4	+1.9	+2.1	+2.2	+2.5
H	-0.5	-0.5	-0.2	-0.4	-0.3	-0.1
I	-1.6	-1.3	+0.6	+0.8	+1.1	+1.5
J	-1.5	-0.7	+2.0	+1.2	+1.3	+1.8

b) 3D synthetic vs. reference CT: PTV Dose Deviations (Green ≤1.0%, Yellow ≤2.0%, Red >2.0%)

VMAT

	$\Delta D_{0.01cc}$	$\Delta D_{2\%}$	$\Delta D_{50\%}$	ΔD_{mean}	$\Delta D_{98\%}$	$\Delta D_{99\%}$
A	+5.0	+5.1	+4.6	+4.6	+3.9	+3.7
B	+4.0	+3.9	+3.6	+3.6	+3.6	+3.4
C	-1.5	-1.3	-1.1	-1.0	-0.9	-0.7
D	+3.1	+3.1	+3.1	+3.1	+2.9	+2.9
E	+3.3	+3.1	+3.0	+3.0	+3.4	+3.3
F	+1.1	+1.5	+1.3	+1.4	+1.5	+1.6
G	+2.6	+2.5	+2.2	+2.3	+2.1	+2.2
H	-0.1	-0.1	-0.1	-0.1	-0.2	-0.2
I	+1.8	+1.9	+1.5	+1.5	+1.0	+1.3
J	+1.9	+2.1	+2.1	+2.1	+2.5	+3.1

APLR

	$\Delta D_{0.01cc}$	$\Delta D_{2\%}$	$\Delta D_{50\%}$	ΔD_{mean}	$\Delta D_{98\%}$	$\Delta D_{99\%}$
A	+4.9	+4.8	+4.3	+4.2	+4.9	+4.5
B	+5.4	+5.4	+5.3	+5.0	+4.7	+4.7
C	+0.1	+0.1	+0.8	+0.4	+0.9	-0.3
D	+3.6	+3.6	+3.7	+3.7	+2.7	+3.0
E	+4.9	+5.0	+5.1	+5.0	+5.9	+5.1
F	+1.6	+2.3	+4.8	+4.1	+13.3	+0.8
G	+1.9	+1.9	+1.6	+1.5	+1.6	+2.2
H	+0.5	+0.4	+0.2	+0.5	+1.2	+0.5
I	+2.7	+2.9	+4.5	+3.8	+3.7	+2.9
J	+8.0	+8.4	+9.5	+8.9	+12.6	+4.9

AP

	$\Delta D_{0.01cc}$	$\Delta D_{2\%}$	$\Delta D_{50\%}$	ΔD_{mean}	$\Delta D_{98\%}$	$\Delta D_{99\%}$
A	+3.4	+3.4	+2.3	+2.5	+3.4	+3.4
B	+5.6	+5.5	+5.5	+5.6	+5.8	+5.7
C	-2.5	-2.5	-2.7	-2.7	-3.0	-3.5
D	+1.7	+1.5	+1.6	+1.6	+1.3	+1.6
E	+6.3	+6.6	+7.5	+7.8	+6.9	+6.8
F	+3.6	+3.6	+5.0	+5.4	+1.2	-0.1
G	+3.1	+3.2	+2.0	+2.5	+3.7	+4.2
H	+0.2	+0.4	+0.2	+0.6	-0.1	+0.1
I	+1.0	+2.7	+3.5	+3.5	+1.4	+3.2
J	+15.2	+15.0	+17.7	+16.6	+13.6	+3.3

PA

	$\Delta D_{0.01cc}$	$\Delta D_{2\%}$	$\Delta D_{50\%}$	ΔD_{mean}	$\Delta D_{98\%}$	$\Delta D_{99\%}$
A	+1.8	+2.0	+2.4	+2.7	+4.5	+1.1
B	+1.9	+2.3	+2.2	+2.2	+1.1	+1.4
C	+0.1	+0.1	+0.2	-0.2	-2.1	-2.5
D	+9.8	+9.5	+9.3	+9.3	+8.0	+6.8
E	+4.2	+5.5	+5.5	+5.6	+5.7	+9.5
F	+1.8	+1.7	+4.6	+3.7	+0.7	+2.0
G	+3.9	+3.8	+3.5	+3.4	+2.3	+3.0
H	+3.0	+2.8	+3.4	+3.3	+3.4	+3.5
I	+5.0	+3.7	+6.7	+6.1	+1.5	+1.4
J	+2.5	+2.9	+7.6	+6.7	+1.8	+4.6

LR

	$\Delta D_{0.01cc}$	$\Delta D_{2\%}$	$\Delta D_{50\%}$	ΔD_{mean}	$\Delta D_{98\%}$	$\Delta D_{99\%}$
A	+11.0	+10.0	+8.3	+8.4	+5.4	+5.5
B	+7.0	+7.5	+8.1	+7.8	+7.4	+6.5
C	+4.8	+4.2	+4.4	+4.2	+5.0	+5.4
D	+2.2	+2.2	+2.0	+2.1	+2.2	+2.4
E	+2.9	+3.2	+2.4	+2.4	+1.1	+1.8
F	+0.1	+0.1	+2.3	+3.2	+1.7	+1.5
G	+0.6	+0.6	+0.9	+0.8	+1.3	+0.9
H	-1.5	-1.4	-1.2	-1.3	-1.3	-1.3
I	+5.2	+5.5	+7.5	+7.6	+5.3	+5.0
J	+6.5	+6.3	+11.6	+9.7	+11.1	+11.3

RL

	$\Delta D_{0.01cc}$	$\Delta D_{2\%}$	$\Delta D_{50\%}$	ΔD_{mean}	$\Delta D_{98\%}$	$\Delta D_{99\%}$
A	+3.7	+3.9	+2.8	+3.2	+4.2	+5.0
B	+6.4	+4.8	+3.5	+4.3	+5.0	+1.7
C	+1.4	+0.7	+0.3	-0.1	-1.9	-1.5
D	+1.9	+1.7	+1.9	+1.8	+1.8	+1.8
E	+3.8	+3.9	+5.2	+4.5	+3.9	+4.2
F	-0.2	-0.3	+3.1	+4.2	+8.3	+2.1
G	+1.0	+0.7	-0.3	-0.4	-4.0	-0.8
H	-0.1	+0.2	+0.0	-0.4	-0.1	-0.3
I	-4.4	-4.5	-1.4	-1.8	+1.1	+0.9
J	-0.6	+0.1	+4.0	+3.6	+3.2	+2.8

Figure S5.1 Thresholds green ≤ 1%, yellow 1–2%, red > 2%.

a) 2D synthetic vs. reference CT: PTV Dose Deviations (Green ≤1.0%, Yellow ≤3.0%, Red >3.0%)

	VMAT						APLR					
	$\Delta D_{0.01cc}$	$\Delta D_{2\%}$	$\Delta D_{50\%}$	ΔD_{mean}	$\Delta D_{98\%}$	$\Delta D_{99\%}$	$\Delta D_{0.01cc}$	$\Delta D_{2\%}$	$\Delta D_{50\%}$	ΔD_{mean}	$\Delta D_{98\%}$	$\Delta D_{99\%}$
A	+3.9	+3.9	+3.8	+3.9	+3.9	+3.8	+3.8	+3.7	+3.5	+3.6	+3.6	+3.6
B	+2.5	+2.3	+2.2	+2.2	+2.2	+2.0	+3.5	+3.2	+3.3	+2.9	+2.6	+2.7
C	-3.2	-3.2	-3.0	-2.9	-2.5	-2.2	-2.8	-2.7	-2.1	-2.3	-2.0	-2.8
D	+3.3	+3.2	+3.3	+3.3	+3.2	+3.2	+3.1	+3.2	+3.1	+3.1	+3.0	+3.2
E	+2.5	+2.5	+2.0	+2.0	+1.5	+1.5	+3.9	+3.8	+3.6	+3.5	+2.8	+1.7
F	+4.6	+4.7	+1.7	+2.1	+1.5	+1.6	+2.7	+3.5	+8.5	+6.5	+12.8	+0.9
G	+1.2	+1.3	+1.1	+1.0	+0.9	+0.8	+1.2	+1.0	+1.1	+1.0	+0.7	+1.1
H	-0.2	-0.2	-0.2	-0.2	-0.2	-0.3	-0.5	-0.5	-0.6	-0.5	-0.3	-0.3
I	+3.3	+3.4	+2.7	+2.5	+1.0	+1.5	+5.0	+5.1	+6.8	+5.6	+4.5	+4.3
J	+0.7	+0.8	+1.1	+1.1	+1.8	+2.5	+3.7	+3.7	+4.6	+4.1	+7.5	+2.8

	AP						PA					
	$\Delta D_{0.01cc}$	$\Delta D_{2\%}$	$\Delta D_{50\%}$	ΔD_{mean}	$\Delta D_{98\%}$	$\Delta D_{99\%}$	$\Delta D_{0.01cc}$	$\Delta D_{2\%}$	$\Delta D_{50\%}$	ΔD_{mean}	$\Delta D_{98\%}$	$\Delta D_{99\%}$
A	+1.1	+1.1	+1.1	+1.1	+1.0	+1.2	+6.7	+6.5	+6.8	+6.8	+6.9	+6.6
B	+6.4	+6.3	+6.3	+6.3	+6.3	+6.0	+0.3	+0.2	+0.3	+0.3	+0.2	+0.3
C	-3.1	-3.2	-3.3	-3.2	-3.2	-4.4	-2.3	-2.4	-2.7	-2.5	-3.0	-3.4
D	+1.6	+1.4	+1.6	+1.5	+1.7	+1.6	+4.5	+4.6	+4.7	+4.7	+4.5	+4.4
E	+1.6	+2.6	+2.0	+2.5	+1.2	+2.0	+5.2	+6.0	+4.4	+4.7	+3.2	+11.7
F	+3.6	+3.9	+8.7	+9.0	+2.3	+1.8	-0.9	-0.5	+4.4	+3.2	-0.7	-0.3
G	-1.3	-1.1	-3.5	-2.0	+2.6	-2.0	+2.9	+2.6	+2.5	+2.3	-0.7	+2.2
H	+0.3	+0.6	+0.2	+0.3	+0.4	+0.5	-0.8	-1.0	-0.7	-0.8	-0.9	-0.7
I	+11.9	+11.8	+8.8	+10.7	+6.5	+9.5	+0.9	+1.0	+4.4	+3.6	+1.1	+1.5
J	+9.5	+9.2	+9.2	+9.1	+5.7	+4.9	+3.9	+3.9	+6.9	+6.1	+1.6	+3.6

	LR						RL					
	$\Delta D_{0.01cc}$	$\Delta D_{2\%}$	$\Delta D_{50\%}$	ΔD_{mean}	$\Delta D_{98\%}$	$\Delta D_{99\%}$	$\Delta D_{0.01cc}$	$\Delta D_{2\%}$	$\Delta D_{50\%}$	ΔD_{mean}	$\Delta D_{98\%}$	$\Delta D_{99\%}$
A	+4.2	+4.1	+4.2	+4.1	+4.1	+4.1	+2.3	+2.5	+2.7	+2.4	+2.0	+2.2
B	+3.0	+3.1	+2.3	+2.4	+2.0	+0.9	+4.5	+3.1	+3.3	+3.4	+3.0	+2.0
C	-2.2	-2.2	-2.1	-2.3	-2.8	-2.5	-0.6	-1.2	-1.1	-1.0	-1.9	-1.7
D	+2.9	+3.0	+2.9	+3.0	+2.9	+3.0	+3.2	+3.0	+3.3	+3.2	+3.3	+3.2
E	+3.6	+3.5	+1.9	+2.2	+1.6	+1.3	+3.9	+4.3	+5.1	+4.4	+3.9	+3.6
F	+0.5	+0.6	+5.9	+6.4	+3.4	+2.4	+0.1	+0.1	+7.2	+7.5	+9.8	+2.6
G	+1.0	+1.2	+0.9	+1.1	+1.5	+1.5	+2.5	+2.4	+1.9	+2.1	+2.2	+2.5
H	-1.0	-1.2	-0.9	-1.0	-0.9	-0.9	-0.5	-0.5	-0.2	-0.4	-0.3	-0.1
I	+4.9	+5.4	+7.7	+7.7	+6.5	+5.6	-1.6	-1.3	+0.6	+0.8	+1.1	+1.5
J	-0.9	-0.9	+2.3	+1.1	+2.8	+2.8	-1.5	-0.7	+2.0	+1.2	+1.3	+1.8

b) 3D synthetic vs. reference CT: PTV Dose Deviations (Green ≤1.0%, Yellow ≤3.0%, Red >3.0%)

	VMAT						APLR					
	$\Delta D_{0.01cc}$	$\Delta D_{2\%}$	$\Delta D_{50\%}$	ΔD_{mean}	$\Delta D_{98\%}$	$\Delta D_{99\%}$	$\Delta D_{0.01cc}$	$\Delta D_{2\%}$	$\Delta D_{50\%}$	ΔD_{mean}	$\Delta D_{98\%}$	$\Delta D_{99\%}$
A	+5.0	+5.1	+4.6	+4.6	+3.9	+3.7	+4.9	+4.8	+4.3	+4.2	+4.9	+4.5
B	+4.0	+3.9	+3.6	+3.6	+3.6	+3.4	+5.4	+5.4	+5.3	+5.0	+4.7	+4.7
C	-1.5	-1.3	-1.1	-1.0	-0.9	-0.7	+0.1	+0.1	+0.8	+0.4	+0.9	-0.3
D	+3.1	+3.1	+3.1	+3.1	+2.9	+2.9	+3.6	+3.6	+3.7	+3.7	+2.7	+3.0
E	+3.3	+3.1	+3.0	+3.0	+3.4	+3.3	+4.9	+5.0	+5.1	+5.0	+5.9	+5.1
F	+1.1	+1.5	+1.3	+1.4	+1.5	+1.6	+1.6	+2.3	+4.8	+4.1	+13.3	+0.8
G	+2.6	+2.5	+2.2	+2.3	+2.1	+2.2	+1.9	+1.9	+1.6	+1.5	+1.6	+2.2
H	-0.1	-0.1	-0.1	-0.1	-0.2	-0.2	+0.5	+0.4	+0.2	+0.5	+1.2	+0.5
I	+1.8	+1.9	+1.5	+1.5	+1.0	+1.3	+2.7	+2.9	+4.5	+3.8	+3.7	+2.9
J	+1.9	+2.1	+2.1	+2.1	+2.5	+3.1	+8.0	+8.4	+9.5	+8.9	+12.6	+4.9

	AP						PA					
	$\Delta D_{0.01cc}$	$\Delta D_{2\%}$	$\Delta D_{50\%}$	ΔD_{mean}	$\Delta D_{98\%}$	$\Delta D_{99\%}$	$\Delta D_{0.01cc}$	$\Delta D_{2\%}$	$\Delta D_{50\%}$	ΔD_{mean}	$\Delta D_{98\%}$	$\Delta D_{99\%}$
A	+3.4	+3.4	+2.3	+2.5	+3.4	+3.4	+1.8	+2.0	+2.4	+2.7	+4.5	+1.1
B	+5.6	+5.5	+5.5	+5.6	+5.8	+5.7	+1.9	+2.3	+2.2	+2.2	+1.1	+1.4
C	-2.5	-2.5	-2.7	-2.7	-3.0	-3.5	+0.1	+0.1	+0.2	-0.2	-2.1	-2.5
D	+1.7	+1.5	+1.6	+1.6	+1.3	+1.6	+9.8	+9.5	+9.3	+9.3	+8.0	+6.8
E	+6.3	+6.6	+7.5	+7.8	+6.9	+6.8	+4.2	+5.5	+5.5	+5.6	+5.7	+9.5
F	+3.6	+3.6	+5.0	+5.4	+1.2	-0.1	+1.8	+1.7	+4.6	+3.7	+0.7	+2.0
G	+3.1	+3.2	+2.0	+2.5	+3.7	+4.2	+3.9	+3.8	+3.5	+3.4	+2.3	+3.0
H	+0.2	+0.4	+0.2	+0.6	-0.1	+0.1	+3.0	+2.8	+3.4	+3.3	+3.4	+3.5
I	+1.0	+2.7	+3.5	+3.5	+1.4	+3.2	+5.0	+3.7	+6.7	+6.1	+1.5	+1.4
J	+15.2	+15.0	+17.7	+16.6	+13.6	+3.3	+2.5	+2.9	+7.6	+6.7	+1.8	+4.6

	LR						RL					
	$\Delta D_{0.01cc}$	$\Delta D_{2\%}$	$\Delta D_{50\%}$	ΔD_{mean}	$\Delta D_{98\%}$	$\Delta D_{99\%}$	$\Delta D_{0.01cc}$	$\Delta D_{2\%}$	$\Delta D_{50\%}$	ΔD_{mean}	$\Delta D_{98\%}$	$\Delta D_{99\%}$
A	+11.0	+10.0	+8.3	+8.4	+5.4	+5.5	+3.7	+3.9	+2.8	+3.2	+4.2	+5.0
B	+7.0	+7.5	+8.1	+7.8	+7.4	+6.5	+6.4	+4.8	+3.5	+4.3	+5.0	+1.7
C	+4.8	+4.2	+4.4	+4.2	+5.0	+5.4	+1.4	+0.7	+0.3	-0.1	-1.9	-1.5
D	+2.2	+2.2	+2.0	+2.1	+2.2	+2.4	+1.9	+1.7	+1.9	+1.8	+1.8	+1.8
E	+2.9	+3.2	+2.4	+2.4	+1.1	+1.8	+3.8	+3.9	+5.2	+4.5	+3.9	+4.2
F	+0.1	+0.1	+2.3	+3.2	+1.7	+1.5	-0.2	-0.3	+3.1	+4.2	+8.3	+2.1
G	+0.6	+0.6	+0.9	+0.8	+1.3	+0.9	+1.0	+0.7	-0.3	-0.4	-4.0	-0.8
H	-1.5	-1.4	-1.2	-1.3	-1.3	-1.3	-0.1	+0.2	+0.0	-0.4	-0.1	-0.3
I	+5.2	+5.5	+7.5	+7.6	+5.3	+5.0	-4.4	-4.5	-1.4	-1.8	+1.1	+0.9
J	+6.5	+6.3	+11.6	+9.7	+11.1	+11.3	-0.6	+0.1	+4.0	+3.6	+3.2	+2.8

Figure S5.2 Thresholds green ≤ 1%, yellow 1–3%, red > 3%

a) 2D synthetic vs. reference CT: PTV Dose Deviations (Green ≤1.0%, Yellow ≤5.0%, Red >5.0%)

	VMAT						APLR						AP					
	$\Delta D_{0.01cc}$	$\Delta D_{2\%}$	$\Delta D_{50\%}$	ΔD_{mean}	$\Delta D_{98\%}$	$\Delta D_{99\%}$	$\Delta D_{0.01cc}$	$\Delta D_{2\%}$	$\Delta D_{50\%}$	ΔD_{mean}	$\Delta D_{98\%}$	$\Delta D_{99\%}$	$\Delta D_{0.01cc}$	$\Delta D_{2\%}$	$\Delta D_{50\%}$	ΔD_{mean}	$\Delta D_{98\%}$	$\Delta D_{99\%}$
**A**	+3.9	+3.9	+3.8	+3.9	+3.9	+3.8	+3.8	+3.7	+3.5	+3.6	+3.6	+3.6	+1.1	+1.1	+1.1	+1.1	+1.0	+1.2
**B**	+2.5	+2.3	+2.2	+2.2	+2.2	+2.0	+3.5	+3.2	+3.3	+2.9	+2.6	+2.7	+6.4	+6.3	+6.3	+6.3	+6.3	+6.0
**C**	-3.2	-3.2	-3.0	-2.9	-2.5	-2.2	-2.8	-2.7	-2.1	-2.3	-2.0	-2.8	-3.1	-3.2	-3.3	-3.2	-3.2	-4.4
**D**	+3.3	+3.2	+3.3	+3.3	+3.2	+3.2	+3.1	+3.2	+3.1	+3.1	+3.0	+3.2	+1.6	+1.4	+1.6	+1.5	+1.7	+1.6
**E**	+2.5	+2.5	+2.0	+2.0	+1.5	+1.5	+3.9	+3.8	+3.6	+3.5	+2.8	+1.7	+1.6	+2.6	+2.0	+2.5	+1.2	+2.0
**F**	+4.6	+4.7	+1.7	+2.1	+1.5	+1.6	+2.7	+3.5	+8.5	+6.5	+12.8	+0.9	+3.6	+3.9	+8.7	+9.0	+2.3	+1.8
**G**	+1.2	+1.3	+1.1	+1.0	+0.9	+0.8	+1.2	+1.0	+1.1	+1.0	+0.7	+1.1	-1.3	-1.1	-3.5	-2.0	+2.6	-2.0
**H**	-0.2	-0.2	-0.2	-0.2	-0.2	-0.3	-0.5	-0.5	-0.6	-0.5	-0.3	-0.3	+0.3	+0.6	+0.2	+0.3	+0.4	+0.5
**I**	+3.3	+3.4	+2.7	+2.5	+1.0	+1.5	+5.0	+5.1	+6.8	+5.6	+4.5	+4.3	+11.9	+11.8	+8.8	+10.7	+6.5	+9.5
**J**	+0.7	+0.8	+1.1	+1.1	+1.8	+2.5	+3.7	+3.7	+4.6	+4.1	+7.5	+2.8	+9.5	+9.2	+9.2	+9.1	+5.7	+4.9

	PA						LR						RL					
	$\Delta D_{0.01cc}$	$\Delta D_{2\%}$	$\Delta D_{50\%}$	ΔD_{mean}	$\Delta D_{98\%}$	$\Delta D_{99\%}$	$\Delta D_{0.01cc}$	$\Delta D_{2\%}$	$\Delta D_{50\%}$	ΔD_{mean}	$\Delta D_{98\%}$	$\Delta D_{99\%}$	$\Delta D_{0.01cc}$	$\Delta D_{2\%}$	$\Delta D_{50\%}$	ΔD_{mean}	$\Delta D_{98\%}$	$\Delta D_{99\%}$
**A**	+6.7	+6.5	+6.8	+6.8	+6.9	+6.6	+4.2	+4.1	+4.2	+4.1	+4.1	+4.1	+2.3	+2.5	+2.7	+2.4	+2.0	+2.2
**B**	+0.3	+0.2	+0.3	+0.3	+0.2	+0.3	+3.0	+3.1	+2.3	+2.4	+2.0	+0.9	+4.5	+3.1	+3.3	+3.4	+3.0	+2.0
**C**	-2.3	-2.4	-2.7	-2.5	-3.0	-3.4	-2.2	-2.2	-2.1	-2.3	-2.8	-2.5	-0.6	-1.2	-1.1	-1.0	-1.9	-1.7
**D**	+4.5	+4.6	+4.7	+4.7	+4.5	+4.4	+2.9	+3.0	+2.9	+3.0	+2.9	+3.0	+3.2	+3.0	+3.3	+3.2	+3.3	+3.2
**E**	+5.2	+6.0	+4.4	+4.7	+3.2	+11.7	+3.6	+3.5	+1.9	+2.2	+1.6	+1.3	+3.9	+4.3	+5.1	+4.4	+3.9	+3.6
**F**	-0.9	-0.5	+4.4	+3.2	-0.7	-0.3	+0.5	+0.6	+5.9	+6.4	+3.4	+2.4	+0.1	+0.1	+7.2	+7.5	+9.8	+2.6
**G**	+2.9	+2.6	+2.5	+2.3	-0.7	+2.2	+1.0	+1.2	+0.9	+1.1	+1.5	+1.5	+2.5	+2.4	+1.9	+2.1	+2.2	+2.5
**H**	-0.8	-1.0	-0.7	-0.8	-0.9	-0.7	-1.0	-1.2	-0.9	-1.0	-0.9	-0.9	-0.5	-0.5	-0.2	-0.4	-0.3	-0.1
**I**	+0.9	+1.0	+4.4	+3.6	+1.1	+1.5	+4.9	+5.4	+7.7	+7.7	+6.5	+5.6	-1.6	-1.3	+0.6	+0.8	+1.1	+1.5
**J**	+3.9	+3.9	+6.9	+6.1	+1.6	+3.6	-0.9	-0.9	+2.3	+1.1	+2.8	+2.8	-1.5	-0.7	+2.0	+1.2	+1.3	+1.8

b) 3D synthetic vs. reference CT: PTV Dose Deviations (Green ≤1.0%, Yellow ≤5.0%, Red >5.0%)

	VMAT						APLR						AP					
	$\Delta D_{0.01cc}$	$\Delta D_{2\%}$	$\Delta D_{50\%}$	ΔD_{mean}	$\Delta D_{98\%}$	$\Delta D_{99\%}$	$\Delta D_{0.01cc}$	$\Delta D_{2\%}$	$\Delta D_{50\%}$	ΔD_{mean}	$\Delta D_{98\%}$	$\Delta D_{99\%}$	$\Delta D_{0.01cc}$	$\Delta D_{2\%}$	$\Delta D_{50\%}$	ΔD_{mean}	$\Delta D_{98\%}$	$\Delta D_{99\%}$
**A**	+5.0	+5.1	+4.6	+4.6	+3.9	+3.7	+4.9	+4.8	+4.3	+4.2	+4.9	+4.5	+3.4	+3.4	+2.3	+2.5	+3.4	+3.4
**B**	+4.0	+3.9	+3.6	+3.6	+3.6	+3.4	+5.4	+5.4	+5.3	+5.0	+4.7	+4.7	+5.6	+5.5	+5.5	+5.6	+5.8	+5.7
**C**	-1.5	-1.3	-1.1	-1.0	-0.9	-0.7	+0.1	+0.1	+0.8	+0.4	+0.9	-0.3	-2.5	-2.5	-2.7	-2.7	-3.0	-3.5
**D**	+3.1	+3.1	+3.1	+3.1	+2.9	+2.9	+3.6	+3.6	+3.7	+3.7	+2.7	+3.0	+1.7	+1.5	+1.6	+1.6	+1.3	+1.6
**E**	+3.3	+3.1	+3.0	+3.0	+3.4	+3.3	+4.9	+5.0	+5.1	+5.0	+5.9	+5.1	+6.3	+6.6	+7.5	+7.8	+6.9	+6.8
**F**	+1.1	+1.5	+1.3	+1.4	+1.5	+1.6	+1.6	+2.3	+4.8	+4.1	+13.3	+0.8	+3.6	+3.6	+5.0	+5.4	+1.2	-0.1
**G**	+2.6	+2.5	+2.2	+2.3	+2.1	+2.2	+1.9	+1.9	+1.6	+1.5	+1.6	+2.2	+3.1	+3.2	+2.0	+2.5	+3.7	+4.2
**H**	-0.1	-0.1	-0.1	-0.1	-0.2	-0.2	+0.5	+0.4	+0.2	+0.5	+1.2	+0.5	+0.2	+0.4	+0.2	+0.6	-0.1	+0.1
**I**	+1.8	+1.9	+1.5	+1.5	+1.0	+1.3	+2.7	+2.9	+4.5	+3.8	+3.7	+2.9	+1.0	+2.7	+3.5	+3.5	+1.4	+3.2
**J**	+1.9	+2.1	+2.1	+2.1	+2.5	+3.1	+8.0	+8.4	+9.5	+8.9	+12.6	+4.9	+15.2	+15.0	+17.7	+16.6	+13.6	+3.3

	PA						LR						RL					
	$\Delta D_{0.01cc}$	$\Delta D_{2\%}$	$\Delta D_{50\%}$	ΔD_{mean}	$\Delta D_{98\%}$	$\Delta D_{99\%}$	$\Delta D_{0.01cc}$	$\Delta D_{2\%}$	$\Delta D_{50\%}$	ΔD_{mean}	$\Delta D_{98\%}$	$\Delta D_{99\%}$	$\Delta D_{0.01cc}$	$\Delta D_{2\%}$	$\Delta D_{50\%}$	ΔD_{mean}	$\Delta D_{98\%}$	$\Delta D_{99\%}$
**A**	+1.8	+2.0	+2.4	+2.7	+4.5	+1.1	+11.0	+10.0	+8.3	+8.4	+5.4	+5.5	+3.7	+3.9	+2.8	+3.2	+4.2	+5.0
**B**	+1.9	+2.3	+2.2	+2.2	+1.1	+1.4	+7.0	+7.5	+8.1	+7.8	+7.4	+6.5	+6.4	+4.8	+3.5	+4.3	+5.0	+1.7
**C**	+0.1	+0.1	+0.2	-0.2	-2.1	-2.5	+4.8	+4.2	+4.4	+4.2	+5.0	+5.4	+1.4	+0.7	+0.3	-0.1	-1.9	-1.5
**D**	+9.8	+9.5	+9.3	+9.3	+8.0	+6.8	+2.2	+2.2	+2.0	+2.1	+2.2	+2.4	+1.9	+1.7	+1.9	+1.8	+1.8	+1.8
**E**	+4.2	+5.5	+5.5	+5.6	+5.7	+9.5	+2.9	+3.2	+2.4	+2.4	+1.1	+1.8	+3.8	+3.9	+5.2	+4.5	+3.9	+4.2
**F**	+1.8	+1.7	+4.6	+3.7	+0.7	+2.0	+0.1	+0.1	+2.3	+3.2	+1.7	+1.5	-0.2	-0.3	+3.1	+4.2	+8.3	+2.1
**G**	+3.9	+3.8	+3.5	+3.4	+2.3	+3.0	+0.6	+0.6	+0.9	+0.8	+1.3	+0.9	+1.0	+0.7	-0.3	-0.4	-4.0	-0.8
**H**	+3.0	+2.8	+3.4	+3.3	+3.4	+3.5	-1.5	-1.4	-1.2	-1.3	-1.3	-1.3	-0.1	+0.2	+0.0	-0.4	-0.1	-0.3
**I**	+5.0	+3.7	+6.7	+6.1	+1.5	+1.4	+5.2	+5.5	+7.5	+7.6	+5.3	+5.0	-4.4	-4.5	-1.4	-1.8	+1.1	+0.9
**J**	+2.5	+2.9	+7.6	+6.7	+1.8	+4.6	+6.5	+6.3	+11.6	+9.7	+11.1	+11.3	-0.6	+0.1	+4.0	+3.6	+3.2	+2.8

Figure S5.3 Thresholds green ≤ 1%, yellow 1–5%, red > 5%.

a) 2D synthetic vs. reference CT: PTV Dose Deviations (Green ≤2.0%, Yellow ≤3.0%, Red >3.0%)

	VMAT						APLR					
	$\Delta D_{0.01cc}$	$\Delta D_{2\%}$	$\Delta D_{50\%}$	ΔD_{mean}	$\Delta D_{98\%}$	$\Delta D_{99\%}$	$\Delta D_{0.01cc}$	$\Delta D_{2\%}$	$\Delta D_{50\%}$	ΔD_{mean}	$\Delta D_{98\%}$	$\Delta D_{99\%}$
A	+3.9	+3.9	+3.8	+3.9	+3.9	+3.8	+3.8	+3.7	+3.5	+3.6	+3.6	+3.6
B	+2.5	+2.3	+2.2	+2.2	+2.2	+2.0	+3.5	+3.2	+3.3	+2.9	+2.6	+2.7
C	-3.2	-3.2	-3.0	-2.9	-2.5	-2.2	-2.8	-2.7	-2.1	-2.3	-2.0	-2.8
D	+3.3	+3.2	+3.3	+3.3	+3.2	+3.2	+3.1	+3.2	+3.1	+3.1	+3.0	+3.2
E	+2.5	+2.5	+2.0	+2.0	+1.5	+1.5	+3.9	+3.8	+3.6	+3.5	+2.8	+1.7
F	+4.6	+4.7	+1.7	+2.1	+1.5	+1.6	+2.7	+3.5	+8.5	+6.5	+12.8	+0.9
G	+1.2	+1.3	+1.1	+1.0	+0.9	+0.8	+1.2	+1.0	+1.1	+1.0	+0.7	+1.1
H	-0.2	-0.2	-0.2	-0.2	-0.2	-0.3	-0.5	-0.5	-0.6	-0.5	-0.3	-0.3
I	+3.3	+3.4	+2.7	+2.5	+1.0	+1.5	+5.0	+5.1	+6.8	+5.6	+4.5	+4.3
J	+0.7	+0.8	+1.1	+1.1	+1.8	+2.5	+3.7	+3.7	+4.6	+4.1	+7.5	+2.8

	AP						PA					
	$\Delta D_{0.01cc}$	$\Delta D_{2\%}$	$\Delta D_{50\%}$	ΔD_{mean}	$\Delta D_{98\%}$	$\Delta D_{99\%}$	$\Delta D_{0.01cc}$	$\Delta D_{2\%}$	$\Delta D_{50\%}$	ΔD_{mean}	$\Delta D_{98\%}$	$\Delta D_{99\%}$
A	+1.1	+1.1	+1.1	+1.1	+1.0	+1.2	+6.7	+6.5	+6.8	+6.8	+6.9	+6.6
B	+6.4	+6.3	+6.3	+6.3	+6.3	+6.0	+0.3	+0.2	+0.3	+0.3	+0.2	+0.3
C	-3.1	-3.2	-3.3	-3.2	-3.2	-4.4	-2.3	-2.4	-2.7	-2.5	-3.0	-3.4
D	+1.6	+1.4	+1.6	+1.5	+1.7	+1.6	+4.5	+4.6	+4.7	+4.7	+4.5	+4.4
E	+1.6	+2.6	+2.0	+2.5	+1.2	+2.0	+5.2	+6.0	+4.4	+4.7	+3.2	+11.7
F	+3.6	+3.9	+8.7	+9.0	+2.3	+1.8	-0.9	-0.5	+4.4	+3.2	-0.7	-0.3
G	-1.3	-1.1	-3.5	-2.0	+2.6	-2.0	+2.9	+2.6	+2.5	+2.3	-0.7	+2.2
H	+0.3	+0.6	+0.2	+0.3	+0.4	+0.5	-0.8	-1.0	-0.7	-0.8	-0.9	-0.7
I	+11.9	+11.8	+8.8	+10.7	+6.5	+9.5	+0.9	+1.0	+4.4	+3.6	+1.1	+1.5
J	+9.5	+9.2	+9.2	+9.1	+5.7	+4.9	+3.9	+3.9	+6.9	+6.1	+1.6	+3.6

	LR						RL					
	$\Delta D_{0.01cc}$	$\Delta D_{2\%}$	$\Delta D_{50\%}$	ΔD_{mean}	$\Delta D_{98\%}$	$\Delta D_{99\%}$	$\Delta D_{0.01cc}$	$\Delta D_{2\%}$	$\Delta D_{50\%}$	ΔD_{mean}	$\Delta D_{98\%}$	$\Delta D_{99\%}$
A	+4.2	+4.1	+4.2	+4.1	+4.1	+4.1	+2.3	+2.5	+2.7	+2.4	+2.0	+2.2
B	+3.0	+3.1	+2.3	+2.4	+2.0	+0.9	+4.5	+3.1	+3.3	+3.4	+3.0	+2.0
C	-2.2	-2.2	-2.1	-2.3	-2.8	-2.5	-0.6	-1.2	-1.1	-1.0	-1.9	-1.7
D	+2.9	+3.0	+2.9	+3.0	+2.9	+3.0	+3.2	+3.0	+3.3	+3.2	+3.3	+3.2
E	+3.6	+3.5	+1.9	+2.2	+1.6	+1.3	+3.9	+4.3	+5.1	+4.4	+3.9	+3.6
F	+0.5	+0.6	+5.9	+6.4	+3.4	+2.4	+0.1	+0.1	+7.2	+7.5	+9.8	+2.6
G	+1.0	+1.2	+0.9	+1.1	+1.5	+1.5	+2.5	+2.4	+1.9	+2.1	+2.2	+2.5
H	-1.0	-1.2	-0.9	-1.0	-0.9	-0.9	-0.5	-0.5	-0.2	-0.4	-0.3	-0.1
I	+4.9	+5.4	+7.7	+7.7	+6.5	+5.6	-1.6	-1.3	+0.6	+0.8	+1.1	+1.5
J	-0.9	-0.9	+2.3	+1.1	+2.8	+2.8	-1.5	-0.7	+2.0	+1.2	+1.3	+1.8

b) 3D synthetic vs. reference CT: PTV Dose Deviations (Green ≤2.0%, Yellow ≤3.0%, Red >3.0%)

	VMAT						APLR					
	$\Delta D_{0.01cc}$	$\Delta D_{2\%}$	$\Delta D_{50\%}$	ΔD_{mean}	$\Delta D_{98\%}$	$\Delta D_{99\%}$	$\Delta D_{0.01cc}$	$\Delta D_{2\%}$	$\Delta D_{50\%}$	ΔD_{mean}	$\Delta D_{98\%}$	$\Delta D_{99\%}$
A	+5.0	+5.1	+4.6	+4.6	+3.9	+3.7	+4.9	+4.8	+4.3	+4.2	+4.9	+4.5
B	+4.0	+3.9	+3.6	+3.6	+3.6	+3.4	+5.4	+5.4	+5.3	+5.0	+4.7	+4.7
C	-1.5	-1.3	-1.1	-1.0	-0.9	-0.7	+0.1	+0.1	+0.8	+0.4	+0.9	-0.3
D	+3.1	+3.1	+3.1	+3.1	+2.9	+2.9	+3.6	+3.6	+3.7	+3.7	+2.7	+3.0
E	+3.3	+3.1	+3.0	+3.0	+3.4	+3.3	+4.9	+5.0	+5.1	+5.0	+5.9	+5.1
F	+1.1	+1.5	+1.3	+1.4	+1.5	+1.6	+1.6	+2.3	+4.8	+4.1	+13.3	+0.8
G	+2.6	+2.5	+2.2	+2.3	+2.1	+2.2	+1.9	+1.9	+1.6	+1.5	+1.6	+2.2
H	-0.1	-0.1	-0.1	-0.1	-0.2	-0.2	+0.5	+0.4	+0.2	+0.5	+1.2	+0.5
I	+1.8	+1.9	+1.5	+1.5	+1.0	+1.3	+2.7	+2.9	+4.5	+3.8	+3.7	+2.9
J	+1.9	+2.1	+2.1	+2.1	+2.5	+3.1	+8.0	+8.4	+9.5	+8.9	+12.6	+4.9

	AP						PA					
	$\Delta D_{0.01cc}$	$\Delta D_{2\%}$	$\Delta D_{50\%}$	ΔD_{mean}	$\Delta D_{98\%}$	$\Delta D_{99\%}$	$\Delta D_{0.01cc}$	$\Delta D_{2\%}$	$\Delta D_{50\%}$	ΔD_{mean}	$\Delta D_{98\%}$	$\Delta D_{99\%}$
A	+3.4	+3.4	+2.3	+2.5	+3.4	+3.4	+1.8	+2.0	+2.4	+2.7	+4.5	+1.1
B	+5.6	+5.5	+5.5	+5.6	+5.8	+5.7	+1.9	+2.3	+2.2	+2.2	+1.1	+1.4
C	-2.5	-2.5	-2.7	-2.7	-3.0	-3.5	+0.1	+0.1	+0.2	-0.2	-2.1	-2.5
D	+1.7	+1.5	+1.6	+1.6	+1.3	+1.6	+9.8	+9.5	+9.3	+9.3	+8.0	+6.8
E	+6.3	+6.6	+7.5	+7.8	+6.9	+6.8	+4.2	+5.5	+5.5	+5.6	+5.7	+9.5
F	+3.6	+3.6	+5.0	+5.4	+1.2	-0.1	+1.8	+1.7	+4.6	+3.7	+0.7	+2.0
G	+3.1	+3.2	+2.0	+2.5	+3.7	+4.2	+3.9	+3.8	+3.5	+3.4	+2.3	+3.0
H	+0.2	+0.4	+0.2	+0.6	-0.1	+0.1	+3.0	+2.8	+3.4	+3.3	+3.4	+3.5
I	+1.0	+2.7	+3.5	+3.5	+1.4	+3.2	+5.0	+3.7	+6.7	+6.1	+1.5	+1.4
J	+15.2	+15.0	+17.7	+16.6	+13.6	+3.3	+2.5	+2.9	+7.6	+6.7	+1.8	+4.6

	LR						RL					
	$\Delta D_{0.01cc}$	$\Delta D_{2\%}$	$\Delta D_{50\%}$	ΔD_{mean}	$\Delta D_{98\%}$	$\Delta D_{99\%}$	$\Delta D_{0.01cc}$	$\Delta D_{2\%}$	$\Delta D_{50\%}$	ΔD_{mean}	$\Delta D_{98\%}$	$\Delta D_{99\%}$
A	+11.0	+10.0	+8.3	+8.4	+5.4	+5.5	+3.7	+3.9	+2.8	+3.2	+4.2	+5.0
B	+7.0	+7.5	+8.1	+7.8	+7.4	+6.5	+6.4	+4.8	+3.5	+4.3	+5.0	+1.7
C	+4.8	+4.2	+4.4	+4.2	+5.0	+5.4	+1.4	+0.7	+0.3	-0.1	-1.9	-1.5
D	+2.2	+2.2	+2.0	+2.1	+2.2	+2.4	+1.9	+1.7	+1.9	+1.8	+1.8	+1.8
E	+2.9	+3.2	+2.4	+2.4	+1.1	+1.8	+3.8	+3.9	+5.2	+4.5	+3.9	+4.2
F	+0.1	+0.1	+2.3	+3.2	+1.7	+1.5	-0.2	-0.3	+3.1	+4.2	+8.3	+2.1
G	+0.6	+0.6	+0.9	+0.8	+1.3	+0.9	+1.0	+0.7	-0.3	-0.4	-4.0	-0.8
H	-1.5	-1.4	-1.2	-1.3	-1.3	-1.3	-0.1	+0.2	+0.0	-0.4	-0.1	-0.3
I	+5.2	+5.5	+7.5	+7.6	+5.3	+5.0	-4.4	-4.5	-1.4	-1.8	+1.1	+0.9
J	+6.5	+6.3	+11.6	+9.7	+11.1	+11.3	-0.6	+0.1	+4.0	+3.6	+3.2	+2.8

Figure S5.4 Thresholds green ≤ 2%, yellow 2–3%, red > 3%.

a) 2D synthetic vs. reference CT: PTV Dose Deviations

VMAT	$\Delta D_{0.01cc}$	$\Delta D_{2\%}$	$\Delta D_{50\%}$	ΔD_{mean}	$\Delta D_{98\%}$	$\Delta D_{99\%}$
A	+3.9	+3.9	+3.8	+3.9	+3.9	+3.8
B	+2.5	+2.3	+2.2	+2.2	+2.2	+2.0
C	-3.2	-3.2	-3.0	-2.9	-2.5	-2.2
D	+3.3	+3.2	+3.3	+3.3	+3.2	+3.2
E	+2.5	+2.5	+2.0	+2.0	+1.5	+1.5
F	+4.6	+4.7	+1.7	+2.1	+1.5	+1.6
G	+1.2	+1.3	+1.1	+1.0	+0.9	+0.8
H	-0.2	-0.2	-0.2	-0.2	-0.2	-0.3
I	+3.3	+3.4	+2.7	+2.5	+1.0	+1.5
J	+0.7	+0.8	+1.1	+1.1	+1.8	+2.5

APLR	$\Delta D_{0.01cc}$	$\Delta D_{2\%}$	$\Delta D_{50\%}$	ΔD_{mean}	$\Delta D_{98\%}$	$\Delta D_{99\%}$
A	+3.8	+3.7	+3.5	+3.6	+3.6	+3.6
B	+3.5	+3.2	+3.3	+2.9	+2.6	+2.7
C	-2.8	-2.7	-2.1	-2.3	-2.0	-2.8
D	+3.1	+3.2	+3.1	+3.1	+3.0	+3.2
E	+3.9	+3.8	+3.6	+3.5	+2.8	+1.7
F	+2.7	+3.5	+8.5	+6.5	+12.8	+0.9
G	+1.2	+1.0	+1.1	+1.0	+0.7	+1.1
H	-0.5	-0.5	-0.6	-0.5	-0.3	-0.3
I	+5.0	+5.1	+6.8	+5.6	+4.5	+4.3
J	+3.7	+3.7	+4.6	+4.1	+7.5	+2.8

AP	$\Delta D_{0.01cc}$	$\Delta D_{2\%}$	$\Delta D_{50\%}$	ΔD_{mean}	$\Delta D_{98\%}$	$\Delta D_{99\%}$
A	+1.1	+1.1	+1.1	+1.1	+1.0	+1.2
B	+6.4	+6.3	+6.3	+6.3	+6.3	+6.0
C	-3.1	-3.2	-3.3	-3.2	-3.2	-4.4
D	+1.6	+1.4	+1.6	+1.5	+1.7	+1.6
E	+1.6	+2.6	+2.0	+2.5	+1.2	+2.0
F	+3.6	+3.9	+8.7	+9.0	+2.3	+1.8
G	-1.3	-1.1	-3.5	-2.0	+2.6	-2.0
H	+0.3	+0.6	+0.2	+0.3	+0.4	+0.5
I	+11.9	+11.8	+8.8	+10.7	+6.5	+9.5
J	+9.5	+9.2	+9.2	+9.1	+5.7	+4.9

PA	$\Delta D_{0.01cc}$	$\Delta D_{2\%}$	$\Delta D_{50\%}$	ΔD_{mean}	$\Delta D_{98\%}$	$\Delta D_{99\%}$
A	+6.7	+6.5	+6.8	+6.8	+6.9	+6.6
B	+0.3	+0.2	+0.3	+0.3	+0.2	+0.3
C	-2.3	-2.4	-2.7	-2.5	-3.0	-3.4
D	+4.5	+4.6	+4.7	+4.7	+4.5	+4.4
E	+5.2	+6.0	+4.4	+4.7	+3.2	+11.7
F	-0.9	-0.5	+4.4	+3.2	-0.7	-0.3
G	+2.9	+2.6	+2.5	+2.3	-0.7	+2.2
H	-0.8	-1.0	-0.7	-0.8	-0.9	-0.7
I	+0.9	+1.0	+4.4	+3.6	+1.1	+1.5
J	+3.9	+3.9	+6.9	+6.1	+1.6	+3.6

LR	$\Delta D_{0.01cc}$	$\Delta D_{2\%}$	$\Delta D_{50\%}$	ΔD_{mean}	$\Delta D_{98\%}$	$\Delta D_{99\%}$
A	+4.2	+4.1	+4.2	+4.1	+4.1	+4.1
B	+3.0	+3.1	+2.3	+2.4	+2.0	+0.9
C	-2.2	-2.2	-2.1	-2.3	-2.8	-2.5
D	+2.9	+3.0	+2.9	+3.0	+2.9	+3.0
E	+3.6	+3.5	+1.9	+2.2	+1.6	+1.3
F	+0.5	+0.6	+5.9	+6.4	+3.4	+2.4
G	+1.0	+1.2	+0.9	+1.1	+1.5	+1.5
H	-1.0	-1.2	-0.9	-1.0	-0.9	-0.9
I	+4.9	+5.4	+7.7	+7.7	+6.5	+5.6
J	-0.9	-0.9	+2.3	+1.1	+2.8	+2.8

RL	$\Delta D_{0.01cc}$	$\Delta D_{2\%}$	$\Delta D_{50\%}$	ΔD_{mean}	$\Delta D_{98\%}$	$\Delta D_{99\%}$
A	+2.3	+2.5	+2.7	+2.4	+2.0	+2.2
B	+4.5	+3.1	+3.3	+3.4	+3.0	+2.0
C	-0.6	-1.2	-1.1	-1.0	-1.9	-1.7
D	+3.2	+3.0	+3.3	+3.2	+3.3	+3.2
E	+3.9	+4.3	+5.1	+4.4	+3.9	+3.6
F	+0.1	+0.1	+7.2	+7.5	+9.8	+2.6
G	+2.5	+2.4	+1.9	+2.1	+2.2	+2.5
H	-0.5	-0.5	-0.2	-0.4	-0.3	-0.1
I	-1.6	-1.3	+0.6	+0.8	+1.1	+1.5
J	-1.5	-0.7	+2.0	+1.2	+1.3	+1.8

b) 3D synthetic vs. reference CT: PTV Dose Deviations

VMAT	$\Delta D_{0.01cc}$	$\Delta D_{2\%}$	$\Delta D_{50\%}$	ΔD_{mean}	$\Delta D_{98\%}$	$\Delta D_{99\%}$
A	+5.0	+5.1	+4.6	+4.6	+3.9	+3.7
B	+4.0	+3.9	+3.6	+3.6	+3.6	+3.4
C	-1.5	-1.3	-1.1	-1.0	-0.9	-0.7
D	+3.1	+3.1	+3.1	+3.1	+2.9	+2.9
E	+3.3	+3.1	+3.0	+3.0	+3.4	+3.3
F	+1.1	+1.5	+1.3	+1.4	+1.5	+1.6
G	+2.6	+2.5	+2.2	+2.3	+2.1	+2.2
H	-0.1	-0.1	-0.1	-0.1	-0.2	-0.2
I	+1.8	+1.9	+1.5	+1.5	+1.0	+1.3
J	+1.9	+2.1	+2.1	+2.1	+2.5	+3.1

APLR	$\Delta D_{0.01cc}$	$\Delta D_{2\%}$	$\Delta D_{50\%}$	ΔD_{mean}	$\Delta D_{98\%}$	$\Delta D_{99\%}$
A	+4.9	+4.8	+4.3	+4.2	+4.9	+4.5
B	+5.4	+5.4	+5.3	+5.0	+4.7	+4.7
C	+0.1	+0.1	+0.8	+0.4	+0.9	-0.3
D	+3.6	+3.6	+3.7	+3.7	+2.7	+3.0
E	+4.9	+5.0	+5.1	+5.0	+5.9	+5.1
F	+1.6	+2.3	+4.8	+4.1	+13.3	+0.8
G	+1.9	+1.9	+1.6	+1.5	+1.6	+2.2
H	+0.5	+0.4	+0.2	+0.5	+1.2	+0.5
I	+2.7	+2.9	+4.5	+3.8	+3.7	+2.9
J	+8.0	+8.4	+9.5	+8.9	+12.6	+4.9

AP	$\Delta D_{0.01cc}$	$\Delta D_{2\%}$	$\Delta D_{50\%}$	ΔD_{mean}	$\Delta D_{98\%}$	$\Delta D_{99\%}$
A	+3.4	+3.4	+2.3	+2.5	+3.4	+3.4
B	+5.6	+5.5	+5.5	+5.6	+5.8	+5.7
C	-2.5	-2.5	-2.7	-2.7	-3.0	-3.5
D	+1.7	+1.5	+1.6	+1.6	+1.3	+1.6
E	+6.3	+6.6	+7.5	+7.8	+6.9	+6.8
F	+3.6	+3.6	+5.0	+5.4	+1.2	-0.1
G	+3.1	+3.2	+2.0	+2.5	+3.7	+4.2
H	+0.2	+0.4	+0.2	+0.6	-0.1	+0.1
I	+1.0	+2.7	+3.5	+3.5	+1.4	+3.2
J	+15.2	+15.0	+17.7	+16.6	+13.6	+3.3

PA	$\Delta D_{0.01cc}$	$\Delta D_{2\%}$	$\Delta D_{50\%}$	ΔD_{mean}	$\Delta D_{98\%}$	$\Delta D_{99\%}$
A	+1.8	+2.0	+2.4	+2.7	+4.5	+1.1
B	+1.9	+2.3	+2.2	+2.2	+1.1	+1.4
C	+0.1	+0.1	+0.2	-0.2	-2.1	-2.5
D	+9.8	+9.5	+9.3	+9.3	+8.0	+6.8
E	+4.2	+5.5	+5.5	+5.6	+5.7	+9.5
F	+1.8	+1.7	+4.6	+3.7	+0.7	+2.0
G	+3.9	+3.8	+3.5	+3.4	+2.3	+3.0
H	+3.0	+2.8	+3.4	+3.3	+3.4	+3.5
I	+5.0	+3.7	+6.7	+6.1	+1.5	+1.4
J	+2.5	+2.9	+7.6	+6.7	+1.8	+4.6

LR	$\Delta D_{0.01cc}$	$\Delta D_{2\%}$	$\Delta D_{50\%}$	ΔD_{mean}	$\Delta D_{98\%}$	$\Delta D_{99\%}$
A	+11.0	+10.0	+8.3	+8.4	+5.4	+5.5
B	+7.0	+7.5	+8.1	+7.8	+7.4	+6.5
C	+4.8	+4.2	+4.4	+4.2	+5.0	+5.4
D	+2.2	+2.2	+2.0	+2.1	+2.2	+2.4
E	+2.9	+3.2	+2.4	+2.4	+1.1	+1.8
F	+0.1	+0.1	+2.3	+3.2	+1.7	+1.5
G	+0.6	+0.6	+0.9	+0.8	+1.3	+0.9
H	-1.5	-1.4	-1.2	-1.3	-1.3	-1.3
I	+5.2	+5.5	+7.5	+7.6	+5.3	+5.0
J	+6.5	+6.3	+11.6	+9.7	+11.1	+11.3

RL	$\Delta D_{0.01cc}$	$\Delta D_{2\%}$	$\Delta D_{50\%}$	ΔD_{mean}	$\Delta D_{98\%}$	$\Delta D_{99\%}$
A	+3.7	+3.9	+2.8	+3.2	+4.2	+5.0
B	+6.4	+4.8	+3.5	+4.3	+5.0	+1.7
C	+1.4	+0.7	+0.3	-0.1	-1.9	-1.5
D	+1.9	+1.7	+1.9	+1.8	+1.8	+1.8
E	+3.8	+3.9	+5.2	+4.5	+3.9	+4.2
F	-0.2	-0.3	+3.1	+4.2	+8.3	+2.1
G	+1.0	+0.7	-0.3	-0.4	-4.0	-0.8
H	-0.1	+0.2	+0.0	-0.4	-0.1	-0.3
I	-4.4	-4.5	-1.4	-1.8	+1.1	+0.9
J	-0.6	+0.1	+4.0	+3.6	+3.2	+2.8

Figure S5.5 Thresholds green ≤ 2%, yellow 2–5%, red > 5%

a) 2D synthetic vs. reference CT: PTV Dose Deviations (Green ≤3.0%, Yellow ≤5.0%, Red >5.0%)

	VMAT						APLR						AP					
	$\Delta D_{0.01cc}$	$\Delta D_{2\%}$	$\Delta D_{50\%}$	ΔD_{mean}	$\Delta D_{98\%}$	$\Delta D_{99\%}$	$\Delta D_{0.01cc}$	$\Delta D_{2\%}$	$\Delta D_{50\%}$	ΔD_{mean}	$\Delta D_{98\%}$	$\Delta D_{99\%}$	$\Delta D_{0.01cc}$	$\Delta D_{2\%}$	$\Delta D_{50\%}$	ΔD_{mean}	$\Delta D_{98\%}$	$\Delta D_{99\%}$
A	+3.9	+3.9	+3.8	+3.9	+3.9	+3.8	+3.8	+3.7	+3.5	+3.6	+3.6	+3.6	+1.1	+1.1	+1.1	+1.1	+1.0	+1.2
B	+2.5	+2.3	+2.2	+2.2	+2.2	+2.0	+3.5	+3.2	+3.3	+2.9	+2.6	+2.7	+6.4	+6.3	+6.3	+6.3	+6.3	+6.0
C	-3.2	-3.2	-3.0	-2.9	-2.5	-2.2	-2.8	-2.7	-2.1	-2.3	-2.0	-2.8	-3.1	-3.2	-3.3	-3.2	-3.2	-4.4
D	+3.3	+3.2	+3.3	+3.3	+3.2	+3.2	+3.1	+3.2	+3.1	+3.1	+3.0	+3.2	+1.6	+1.4	+1.6	+1.5	+1.7	+1.6
E	+2.5	+2.5	+2.0	+2.0	+1.5	+1.5	+3.9	+3.8	+3.6	+3.5	+2.8	+1.7	+1.6	+2.6	+2.0	+2.5	+1.2	+2.0
F	+4.6	+4.7	+1.7	+2.1	+1.5	+1.6	+2.7	+3.5	+8.5	+6.5	+12.8	+0.9	+3.6	+3.9	+8.7	+9.0	+2.3	+1.8
G	+1.2	+1.3	+1.1	+1.0	+0.9	+0.8	+1.2	+1.0	+1.1	+1.0	+0.7	+1.1	-1.3	-1.1	-3.5	-2.0	+2.6	-2.0
H	-0.2	-0.2	-0.2	-0.2	-0.2	-0.3	-0.5	-0.5	-0.6	-0.5	-0.3	-0.3	+0.3	+0.6	+0.2	+0.3	+0.4	+0.5
I	+3.3	+3.4	+2.7	+2.5	+1.0	+1.5	+5.0	+5.1	+6.8	+5.6	+4.5	+4.3	+11.9	+11.8	+8.8	+10.7	+6.5	+9.5
J	+0.7	+0.8	+1.1	+1.1	+1.8	+2.5	+3.7	+3.7	+4.6	+4.1	+7.5	+2.8	+9.5	+9.2	+9.2	+9.1	+5.7	+4.9

	PA						LR						RL					
	$\Delta D_{0.01cc}$	$\Delta D_{2\%}$	$\Delta D_{50\%}$	ΔD_{mean}	$\Delta D_{98\%}$	$\Delta D_{99\%}$	$\Delta D_{0.01cc}$	$\Delta D_{2\%}$	$\Delta D_{50\%}$	ΔD_{mean}	$\Delta D_{98\%}$	$\Delta D_{99\%}$	$\Delta D_{0.01cc}$	$\Delta D_{2\%}$	$\Delta D_{50\%}$	ΔD_{mean}	$\Delta D_{98\%}$	$\Delta D_{99\%}$
A	+6.7	+6.5	+6.8	+6.8	+6.9	+6.6	+4.2	+4.1	+4.2	+4.1	+4.1	+4.1	+2.3	+2.5	+2.7	+2.4	+2.0	+2.2
B	+0.3	+0.2	+0.3	+0.3	+0.2	+0.3	+3.0	+3.1	+2.3	+2.4	+2.0	+0.9	+4.5	+3.1	+3.3	+3.4	+3.0	+2.0
C	-2.3	-2.4	-2.7	-2.5	-3.0	-3.4	-2.2	-2.2	-2.1	-2.3	-2.8	-2.5	-0.6	-1.2	-1.1	-1.0	-1.9	-1.7
D	+4.5	+4.6	+4.7	+4.7	+4.5	+4.4	+2.9	+3.0	+2.9	+3.0	+2.9	+3.0	+3.2	+3.0	+3.3	+3.2	+3.3	+3.2
E	+5.2	+6.0	+4.4	+4.7	+3.2	+11.7	+3.6	+3.5	+1.9	+2.2	+1.6	+1.3	+3.9	+4.3	+5.1	+4.4	+3.9	+3.6
F	-0.9	-0.5	+4.4	+3.2	-0.7	-0.3	+0.5	+0.6	+5.9	+6.4	+3.4	+2.4	+0.1	+0.1	+7.2	+7.5	+9.8	+2.6
G	+2.9	+2.6	+2.5	+2.3	-0.7	+2.2	+1.0	+1.2	+0.9	+1.1	+1.5	+1.5	+2.5	+2.4	+1.9	+2.1	+2.2	+2.5
H	-0.8	-1.0	-0.7	-0.8	-0.9	-0.7	-1.0	-1.2	-0.9	-1.0	-0.9	-0.9	-0.5	-0.5	-0.2	-0.4	-0.3	-0.1
I	+0.9	+1.0	+4.4	+3.6	+1.1	+1.5	+4.9	+5.4	+7.7	+7.7	+6.5	+5.6	-1.6	-1.3	+0.6	+0.8	+1.1	+1.5
J	+3.9	+3.9	+6.9	+6.1	+1.6	+3.6	-0.9	-0.9	+2.3	+1.1	+2.8	+2.8	-1.5	-0.7	+2.0	+1.2	+1.3	+1.8

b) 3D synthetic vs. reference CT: PTV Dose Deviations (Green ≤3.0%, Yellow ≤5.0%, Red >5.0%)

	VMAT						APLR						AP					
	$\Delta D_{0.01cc}$	$\Delta D_{2\%}$	$\Delta D_{50\%}$	ΔD_{mean}	$\Delta D_{98\%}$	$\Delta D_{99\%}$	$\Delta D_{0.01cc}$	$\Delta D_{2\%}$	$\Delta D_{50\%}$	ΔD_{mean}	$\Delta D_{98\%}$	$\Delta D_{99\%}$	$\Delta D_{0.01cc}$	$\Delta D_{2\%}$	$\Delta D_{50\%}$	ΔD_{mean}	$\Delta D_{98\%}$	$\Delta D_{99\%}$
A	+5.0	+5.1	+4.6	+4.6	+3.9	+3.7	+4.9	+4.8	+4.3	+4.2	+4.9	+4.5	+3.4	+3.4	+2.3	+2.5	+3.4	+3.4
B	+4.0	+3.9	+3.6	+3.6	+3.6	+3.4	+5.4	+5.4	+5.3	+5.0	+4.7	+4.7	+5.6	+5.5	+5.5	+5.6	+5.8	+5.7
C	-1.5	-1.3	-1.1	-1.0	-0.9	-0.7	+0.1	+0.1	+0.8	+0.4	+0.9	-0.3	-2.5	-2.5	-2.7	-2.7	-3.0	-3.5
D	+3.1	+3.1	+3.1	+3.1	+2.9	+2.9	+3.6	+3.6	+3.7	+3.7	+2.7	+3.0	+1.7	+1.5	+1.6	+1.6	+1.3	+1.6
E	+3.3	+3.1	+3.0	+3.0	+3.4	+3.3	+4.9	+5.0	+5.1	+5.0	+5.9	+5.1	+6.3	+6.6	+7.5	+7.8	+6.9	+6.8
F	+1.1	+1.5	+1.3	+1.4	+1.5	+1.6	+1.6	+2.3	+4.8	+4.1	+13.3	+0.8	+3.6	+3.6	+5.0	+5.4	+1.2	-0.1
G	+2.6	+2.5	+2.2	+2.3	+2.1	+2.2	+1.9	+1.9	+1.6	+1.5	+1.6	+2.2	+3.1	+3.2	+2.0	+2.5	+3.7	+4.2
H	-0.1	-0.1	-0.1	-0.1	-0.2	-0.2	+0.5	+0.4	+0.2	+0.5	+1.2	+0.5	+0.2	+0.4	+0.2	+0.6	-0.1	+0.1
I	+1.8	+1.9	+1.5	+1.5	+1.0	+1.3	+2.7	+2.9	+4.5	+3.8	+3.7	+2.9	+1.0	+2.7	+3.5	+3.5	+1.4	+3.2
J	+1.9	+2.1	+2.1	+2.1	+2.5	+3.1	+8.0	+8.4	+9.5	+8.9	+12.6	+4.9	+15.2	+15.0	+17.7	+16.6	+13.6	+3.3

	PA						LR						RL					
	$\Delta D_{0.01cc}$	$\Delta D_{2\%}$	$\Delta D_{50\%}$	ΔD_{mean}	$\Delta D_{98\%}$	$\Delta D_{99\%}$	$\Delta D_{0.01cc}$	$\Delta D_{2\%}$	$\Delta D_{50\%}$	ΔD_{mean}	$\Delta D_{98\%}$	$\Delta D_{99\%}$	$\Delta D_{0.01cc}$	$\Delta D_{2\%}$	$\Delta D_{50\%}$	ΔD_{mean}	$\Delta D_{98\%}$	$\Delta D_{99\%}$
A	+1.8	+2.0	+2.4	+2.7	+4.5	+1.1	+11.0	+10.0	+8.3	+8.4	+5.4	+5.5	+3.7	+3.9	+2.8	+3.2	+4.2	+5.0
B	+1.9	+2.3	+2.2	+2.2	+1.1	+1.4	+7.0	+7.5	+8.1	+7.8	+7.4	+6.5	+6.4	+4.8	+3.5	+4.3	+5.0	+1.7
C	+0.1	+0.1	+0.2	-0.2	-2.1	-2.5	+4.8	+4.2	+4.4	+4.2	+5.0	+5.4	+1.4	+0.7	+0.3	-0.1	-1.9	-1.5
D	+9.8	+9.5	+9.3	+9.3	+8.0	+6.8	+2.2	+2.2	+2.0	+2.1	+2.2	+2.4	+1.9	+1.7	+1.9	+1.8	+1.8	+1.8
E	+4.2	+5.5	+5.5	+5.6	+5.7	+9.5	+2.9	+3.2	+2.4	+2.4	+1.1	+1.8	+3.8	+3.9	+5.2	+4.5	+3.9	+4.2
F	+1.8	+1.7	+4.6	+3.7	+0.7	+2.0	+0.1	+0.1	+2.3	+3.2	+1.7	+1.5	-0.2	-0.3	+3.1	+4.2	+8.3	+2.1
G	+3.9	+3.8	+3.5	+3.4	+2.3	+3.0	+0.6	+0.6	+0.9	+0.8	+1.3	+0.9	+1.0	+0.7	-0.3	-0.4	-4.0	-0.8
H	+3.0	+2.8	+3.4	+3.3	+3.4	+3.5	-1.5	-1.4	-1.2	-1.3	-1.3	-1.3	-0.1	+0.2	+0.0	-0.4	-0.1	-0.3
I	+5.0	+3.7	+6.7	+6.1	+1.5	+1.4	+5.2	+5.5	+7.5	+7.6	+5.3	+5.0	-4.4	-4.5	-1.4	-1.8	+1.1	+0.9
J	+2.5	+2.9	+7.6	+6.7	+1.8	+4.6	+6.5	+6.3	+11.6	+9.7	+11.1	+11.3	-0.6	+0.1	+4.0	+3.6	+3.2	+2.8

Figure S5.6 Thresholds green ≤ 3%, yellow 3–5%, red > 5%.
